# Systematic identification and characterization of exon–intron circRNAs

**DOI:** 10.1101/gr.278590.123

**Published:** 2024-03

**Authors:** Yinchun Zhong, Yan Yang, Xiaolin Wang, Bingbing Ren, Xueren Wang, Ge Shan, Liang Chen

**Affiliations:** 1 Department of Cardiology, The First Affiliated Hospital of USTC, Division of Life Science and Medicine, University of Science and Technology of China, Hefei 230027, China;; 2 Hefei National Laboratory for Physical Sciences at Microscale, Department of Clinical Laboratory, The First Affiliated Hospital of USTC, School of Basic Medical Sciences, Division of Life Science and Medicine, University of Science and Technology of China, Hefei 230027, China;; 3 Department of Pulmonary and Critical Care Medicine, Regional Medical Center for National Institute of Respiratory Diseases, Sir Run Run Shaw Hospital, School of Medicine, Zhejiang University, Hangzhou 310016, China;; 4 Department of Anesthesiology, Shanxi Bethune Hospital, Taiyuan 030032, China;; 5 Department of Anesthesiology, Tongji Hospital, Tongji Medical College, Huazhong University of Science and Technology, Wuhan 430030, China

## Abstract

Exon–intron circRNAs (EIciRNAs) are a circRNA subclass with retained introns. Global features of EIciRNAs remain largely unexplored, mainly owing to the lack of bioinformatic tools. The regulation of intron retention (IR) in EIciRNAs and the associated functionality also require further investigation. We developed a framework, FEICP, which efficiently detected EIciRNAs from high-throughput sequencing (HTS) data. EIciRNAs are distinct from exonic circRNAs (EcircRNAs) in aspects such as with larger length, localization in the nucleus, high tissue specificity, and enrichment mostly in the brain. Deep learning analyses revealed that compared with regular introns, the retained introns of circRNAs (CIRs) are shorter in length, have weaker splice site strength, and have higher GC content. Compared with retained introns in linear RNAs (LIRs), CIRs are more likely to form secondary structures and show greater sequence conservation. CIRs are closer to the 5′-end, whereas LIRs are closer to the 3′-end of transcripts. EIciRNA-generating genes are more actively transcribed and associated with epigenetic marks of gene activation. Computational analyses and genome-wide CRISPR screening revealed that SRSF1 binds to CIRs and inhibits the biogenesis of most EIciRNAs. SRSF1 regulates the biogenesis of *EIciLIMK1*, which enhances the expression of *LIMK1* in *cis* to boost neuronal differentiation, exemplifying EIciRNA physiological function. Overall, our study has developed the FEICP pipeline to identify EIciRNAs from HTS data, and reveals multiple features of CIRs and EIciRNAs. SRSF1 has been identified to regulate EIciRNA biogenesis. EIciRNAs and the regulation of EIciRNA biogenesis play critical roles in neuronal differentiation.

Circular RNAs (circRNAs) encoded by the nuclear genome are a large class of covalently closed single-stranded RNAs generated by splicing-related mechanisms (Kristensen et al. 2019; Chen et al. 2022a). Two subclasses of circRNAs, exonic circRNAs (EcircRNAs) and exon–intron circRNAs (EIciRNAs) are generated by backsplicing, which is a form of noncanonical alternative splicing (AS) (Salzman et al. 2012; Li et al. 2015; Sibley et al. 2016; Chen et al. 2022a). In backsplicing, the 3′-end of an exon links to an upstream 5′-end of an exon to form a backsplicing junction (BSJ) and produce a circRNA (Jeck et al. 2013; Guo et al. 2014; Kristensen et al. 2019). EcircRNAs and EIciRNAs are distinct in sequence composition, as EIciRNAs have retained introns whereas EcircRNAs contain exonic sequences exclusively (Jeck et al. 2013; Guo et al. 2014; Li et al. 2015).

Several computational tools have been developed to detect circRNAs from high-throughput sequencing (HTS) data (Hansen et al. 2016; Zeng et al. 2017). Essentially all these methods are based on searching for BSJs, whereas internal sequences of circRNAs are neglected. There are already several attempts to reconstruct full-length circRNAs from HTS reads (Gao et al. 2016; Wu et al. 2019; Zheng et al. 2019; Yu et al. 2021; Stefanov and Meyer 2023). In one early study, a pipeline termed CIRI-AS was developed to characterize AS events within circRNAs from HTS data, and only 23 EIciRNAs were detected via CIRI-AS in ENCODE HTS data of the nuclei of five cell lines (Gao et al. 2016). CIRI-full, which combines reverse-overlap (RO) and BSJ features to reconstruct the full-length sequence of circRNAs, can be used to identify exonic, intronic, and intergenic circRNAs (Zheng et al. 2019). CYCLeR constructs a splice graph based on the alignment and can also capture the full-length sequences of circRNAs (Stefanov and Meyer 2023). Oxford Nanopore Technology (ONT) sequencing has also been applied to detect full-length circRNAs in several studies (Liu et al. 2021; Rahimi et al. 2021; Xin et al. 2021; Zhang et al. 2021). For example, ONT sequencing identified 720 EIciRNAs in 12 human tissues and the HEK293 cell line (Xin et al. 2021). Additionally, two recently published databases, circAtlas 3.0 and FL-circAS, collected several million full-length circRNAs from published ONT data (Chiang et al. 2024; Wu et al. 2024). However, HTS has been and is still the primary way to assess genome-wide circRNA profiles, and a reliable pipeline in detecting EIciRNAs from HTS data remains in demand.

A series of studies has been performed to investigate the regulation of backsplicing and the biogenesis of circRNAs (Ivanov et al. 2015; Kristensen et al. 2019; Knupp et al. 2021; Rogalska et al. 2023). Backsplicing is facilitated by reverse complementary sequences in the flanking introns of circularizing exons and RNA-binding proteins (RBPs), such as Muscleblind and Quaking (Ashwal-Fluss et al. 2014; Conn et al. 2015; Li et al. 2015; Xu et al. 2021). Several RBPs, including ADAR and DHX9, have also been found to inhibit backsplicing (Ivanov et al. 2015; Rybak-Wolf et al. 2015; Aktaş et al. 2017). These RBPs bind to specific motifs or inverted complementary sequences in flanking introns to regulate backsplicing. However, almost all related studies have focused on the regulation of backsplicing and thus far have not examined the regulation of IR in EIciRNAs; thus, little is known about the modulation of the production of EIciRNAs versus that of EcircRNAs during backsplicing.

As a type of AS, IR in linear transcripts is widespread in the transcriptome and can regulate gene expression in mammalian cells (Braunschweig et al. 2014; Monteuuis et al. 2019; Yeom et al. 2021; Wong and Schmitz 2022). The major features and physiological roles of IR in linear transcripts (or linear IR [LIR]) are known, and much of the knowledge about LIRs has come from the HTS of poly(A)-plus RNA samples (Braunschweig et al. 2014; Middleton et al. 2017; Yeom et al. 2021). For instance, the introns retained in LIRs have distinct features from those spliced out, such as shorter length, higher GC content, and weaker splice site (SS) strength (Braunschweig et al. 2014; Yeom et al. 2021). Neural cells have more prevalent LIRs than do other cell types (Braunschweig et al. 2014). A genome-wide analysis revealed that the LIRs progressively increase during in vitro neuronal differentiation (Braunschweig et al. 2014). Moreover, the genes with increased LIRs tended to have decreased expression levels and were enriched in Gene Ontology (GO) terms irrelevant to neuronal activity, indicating that LIRs may down-regulate nonneuronal transcripts (Braunschweig et al. 2014).

Unlike in LIR studies, there has been no systematic analysis of the introns retained in EIciRNAs (circRNA intron retention [CIRs]). With respect to the molecular function of EIciRNAs, EIciRNAs localize predominantly to the nucleus and play *cis* roles in enhancing the transcription of their parental genes (Li et al. 2015; Hu and Zhou 2018; Xu et al. 2023). Two EIciRNA members, *EIciEIF3J* and *EIciPAIP2*, can recruit U1 snRNP with the 5′ SS of the retained intron to promote transcriptional initiation (Li et al. 2015).

To investigate EIciRNAs further, we developed a reliable computational pipeline for detecting EIciRNAs from HTS data and revealed their sequence and expression features in this study. We sought to characterize the functional relevance of EIciRNAs and regulations on EIciRNA biogenesis using bioinformatic analyses and experimental evaluations.

## Results

### The FEICP pipeline can be used to effectively identify EIciRNAs from HTS data

**Figure 1.**
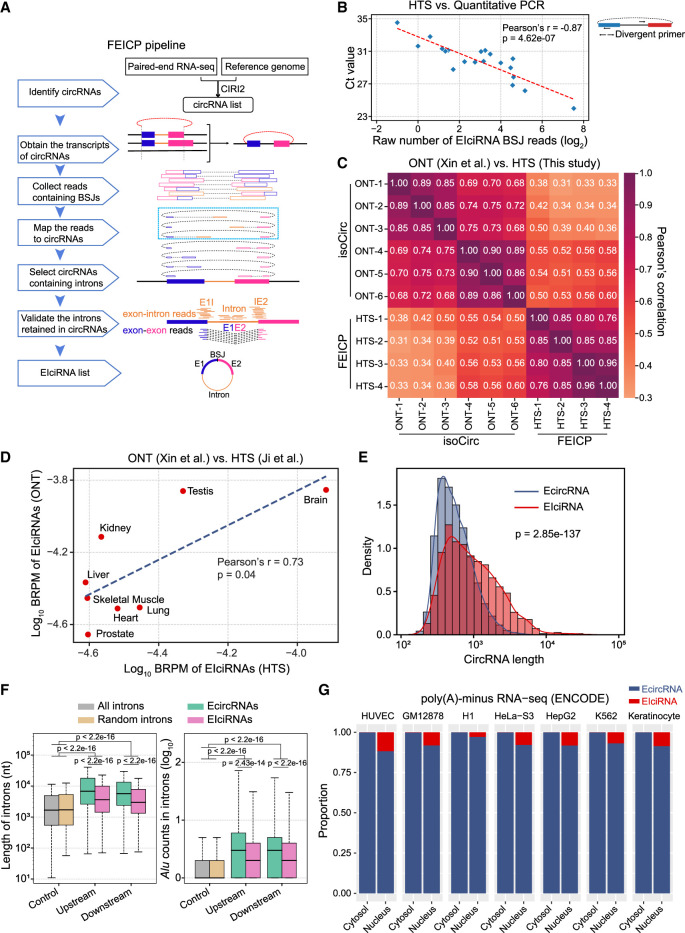
FEICP pipeline and genome-wide analyses of EIciRNAs. (*A*) Workflow of the pipeline finding EIciRNAs from paired-end RNA sequencing (FEICP). Briefly, circRNAs were identified from paired-end high-throughput sequencing (HTS) data, followed by detecting exon–intron junctions or intron sequences from paired reads of backsplicing junctions (BSJs), and then retained introns and EIciRNAs were validated. (*B*) Correlation of Ct value examined by RT-qPCR and BSJ reads predicted by FEICP for 20 randomly selected EIciRNAs in HEK293 cells (*left*). Divergent primers to amplify EIciRNAs were indicated (*right*). (*C*) Pearson’s correlation of the BSJ counts of EIciRNAs commonly detected by HTS and Oxford Nanopore Technology (ONT) sequencing. (*D*) Correlation of EIciRNA BSJ reads (backspliced reads per million [BRPM]) from published HTS or ONT data sets of eight human tissues. (*E*) Distribution of full-length of EcircRNAs and EIciRNAs in HEK293 cells. The full-length of EcircRNAs and EIciRNAs was calculated by psirc and FEICP, respectively. (*F*) Boxplots showing the length of flanking introns of EcircRNAs and EIciRNAs (*left*) and *Alu* counts in their flanking introns (*right*). All introns annotated in the human genome and 2000 randomly selected introns were used as controls. (*G*) Proportion of EcircRNAs and EIciRNAs in cytosol and nucleus across seven human cell lines. For *E* and *F*, *P*-values were calculated using the Wilcoxon rank-sum test.

To identify EIciRNAs from HTS data, we developed finding EIciRNAs from paired-end RNA sequencing (FEICP) (Methods) (Fig. 1A). The first step of FEICP was to detect circRNAs from paired-end RNA sequencing (RNA-seq) data using the CIRI2 pipeline, a sensitive and accurate tool commonly used to identify circRNAs generated by backsplicing (Hansen et al. 2016; Zeng et al. 2017; Vromman et al. 2023). We used paired-end, but not single-end, RNA-seq reads from the FEICP pipeline, as paired-end RNA-seq covers longer regions and would be more reliable in detecting EIciRNAs. Next, based on the BSJs, the annotated host transcripts of the circRNAs were identified, and the HTS reads containing both BSJs and intronic sequences between circularized exons were used to identify candidate EIciRNAs. Selection of HTS reads containing both intronic sequences and BSJs ruled out the possibility of including intronic sequences from linear transcripts. In the bioinformatic identification of LIRs, introns are considered to be retained only when the following criteria are met in the HTS reads: the detection of exon–intron and exon–exon junctions and >90% read coverage of intronic sequences (Braunschweig et al. 2014; Middleton et al. 2017). The read coverage was calculated using all the sequencing reads, as the fragment length of BSJ reads from HTS is limited and generally cannot cover the whole intron. We then applied the same criteria for intron retention (IR) to detect EIciRNAs from the circRNAs (Fig. 1A).

We assessed the EIciRNAs from sequencing reads with different fragment lengths and read lengths (Supplemental Fig. S1A,B). The number and length of detected EIciRNAs increased as the fragment size increased; however, the difference in the number of EIciRNAs among different fragment sizes was relatively small, and the range of EIciRNA lengths was similar across the various fragment sizes of HTS sequencing (Supplemental Fig. S1A). The number of EIciRNAs increased with increasing read length, whereas the increase was small when the read length reached 150 bp. The distribution of EIciRNA lengths remained similar across different read lengths (Supplemental Fig. S1B). We recommend constructing libraries with fragment sizes >250 bp and sequencing with read lengths no less than 150 bp to detect EIciRNAs. Using FEICP, we analyzed four biological replicates of paired-end HTS data from HEK293 cells (Supplemental Table S1). We randomly selected 20 FEICP-identified EIciRNAs and quantified them using RT-qPCR (Fig. 1B; Supplemental Table S2). The results showed that the Ct values and counts of BSJs of EIciRNAs were strongly correlated (Pearson’s correlation = −0.87) (Fig. 1B; Supplemental Table S2). In comparison, CIRI-full identified 287 EIciRNAs, ∼71% (203) of which overlapped with FEICP (Supplemental Fig. S1C). One of the key differences between CIRI-full and FEICP was that CIRI-full was based on BSJ and RO featured to reconstruct full-length circRNAs; thus, the overall length of the EIciRNAs captured by CIRI-full (median length 343 nt) was obviously shorter than that captured by FEICP (median length 821 nt) (Supplemental Fig. S1D).

We next examined the performance of the FEICP pipeline in distinguishing between IR events and cryptic exons. It was found that the proportion of EIciRNAs overlapping with circRNAs containing cryptic exons was similar between FEICP and previous ONT data also from HEK293 cells (isoCirc) (Supplemental Fig. S1E). Out of the total 1824 EIciRNAs, there were 1001 EIciRNAs with exons greater than two; although larger exons might be missed, even they might be included in the EIciRNAs. FEICP was unable to predict EIciRNAs with multiple introns, presumably owing to the relatively larger length of introns. FEICP was unable to detect exons or introns far away from the BSJ; even they might be included in the EIciRNAs. Another pipeline, CYCLeR, identified 2104 EIciRNAs in total, among which 130 and 64 overlapped with FEICP and isoCirc, respectively (Supplemental Fig. S1F). CYCLeR identified circRNA-specific features by comparing circRNA-enriched samples with control total RNA-seq samples, followed by inferring full-length circRNAs using a graph-based algorithm (Stefanov and Meyer 2023). This approach allowed CYCLeR to detect IR in circRNAs independently of BSJ reads, and identified EIciRNAs with a larger length spanning more exons and introns compared with the other three pipelines (Supplemental Fig. S1D,G). The distinct algorithm of CYCLeR could account for the relatively small overlap in EIciRNA identification between CYCLeR and the other software (Supplemental Fig. S1F). Comparable numbers of EIciRNAs, circRNAs, and proportions of BSJ reads were detected between our HTS data and isoCirc data, and 348 EIciRNAs were shared from FEICP identification and isoCirc (Supplemental Figs. S1F, S2A; Supplemental Table S1; Xin et al. 2021). Similar to the approach used by circtools to infer exon inclusion in circRNAs, we extracted the full-length of circRNAs detected by isoCirc, followed by quantifying their abundance in our RNase R+ HTS data and public RNase R– HTS data (Jakobi et al. 2019). It was found that ∼70% of all circRNAs and ∼75% of EIciRNAs were enriched significantly by RNase R treatment, and the proportion of enrichment increased as the BSJ count increased, indicating a good agreement of internal exon–intron structures of circRNAs between the two data sets (Supplemental Fig. S2B). Detection of circRNAs by isoCirc was dependent on rolling circle, which might be prone to miss relatively longer EIciRNAs, and it was noticed that isoCirc-detected EIciRNAs had a shorter length compared with those identified by FEICP (Supplemental Fig. S1D). Among 317 out of 343 EIciRNAs with larger lengths (1500–4000 nt) whose expression was detected by FEICP but not by isoCirc, we successfully validated 10 EIciRNAs with the highest BSJ reads via RT-PCR experiment (Supplemental Fig. S2C; Supplemental Table S2). We compared the correlation of the expression levels (the BSJ counts) of the 348 overlapping EIciRNAs and found that the correlation among four HTS replicates was 0.76–0.96, and the correlation between the HTS data and isoCirc was 0.31–0.60 (Fig. 1C), indicating the reliability of FEICP in quantifying EIciRNAs.

Next, we detected EIciRNAs in 17 human tissues using FEICP in a published study (Ji et al. 2019). EIciRNAs of eight out of these 17 tissues were also characterized in isoCirc data (Xin et al. 2021). We found that the correlation between the number of EIciRNAs in the eight tissues from the two data sets was 0.88 (Supplemental Fig. S2D), and the correlation between the normalized number of EIciRNA BSJs between these data sets was 0.73 (Fig. 1D). Taken together, these findings suggest that FEICP is reliable for identifying EIciRNAs from paired-end HTS data.

### Features of EIciRNAs compared with those of EcircRNAs

A total of 1824 EIciRNAs and 14,445 EcircRNAs were identified in HEK293 cells (Supplemental Fig. S3A). Most of the EIciRNAs (1771, 97.09%) and EcircRNAs (14,165, 98.06%) were generated from protein-coding genes (PCGs) (Supplemental Fig. S3A). Among the 13,510 expressed PCGs in HEK293 cells, 9.81% (1326) could generate EIciRNAs, and 91.63% of EIciRNA-generating genes produced only one or two isoforms of EIciRNAs (Supplemental Fig. S3B). In comparison, 37.69% (5092) of the genes could generate EcircRNAs, and 63.75% of the EcircRNA-generating genes produced one or two isoforms of EcircRNAs (Supplemental Fig. S3C).

We next compared the sequence features of EIciRNAs and EcircRNAs. The length of the EIciRNAs (median length of 821 nt) was significantly longer than that of the EcircRNAs (median length of 512 nt) (Fig. 1E), which was reasonable considering that the EIciRNAs harbored retained introns. The flanking introns of both the EcircRNAs and EIciRNAs were significantly longer and contained more *Alu* elements than did the controls; these introns were all introns in the human genome or 2000 randomly selected introns (Fig. 1F). Previous studies have revealed that longer and more *Alu* sequences in flanking introns are features of backsplicing-generated circRNAs (Jeck et al. 2013; Westholm et al. 2014; Li et al. 2015). The flanking introns of EIciRNAs were significantly shorter and had fewer *Alu* elements than did those of EcircRNAs (Fig. 1F). EIciRNAs and EcircRNAs had stronger strength than the controls of random introns for both of the 3′ SSs of the upstream flanking introns and the 5′ SS of the downstream flanking introns (Supplemental Fig. S3D), whereas the strength of either the 5′ SS of the upstream flanking introns or the 3′ SS of the downstream flanking introns of the EIciRNAs and EcircRNAs was not significantly different (Supplemental Fig. S3D). Furthermore, the 3′ SS strength of the upstream flanking introns of EIciRNAs was significantly greater than that of EcircRNAs (Supplemental Fig. S3D).

To characterize the transcriptome-wide cellular localization of the EIciRNAs and EcircRNAs, we applied FEICP and CIRI2 to the nuclear and cytoplasmic poly(A)-minus RNA-seq data of seven cell lines from The ENCODE Project (The ENCODE Project Consortium 2012) to detect EIciRNAs and EcircRNAs, respectively (Fig. 1G). In these seven cell lines, essentially all the EIciRNAs were localized in the nucleus (Fig. 1G). In addition to showing the innate property of CIR, EIciRNAs displayed several features distinct from those of EcircRNAs.

### The expression of EIciRNAs is highly tissue specific and finely regulated by RBPs

**Figure 2.**
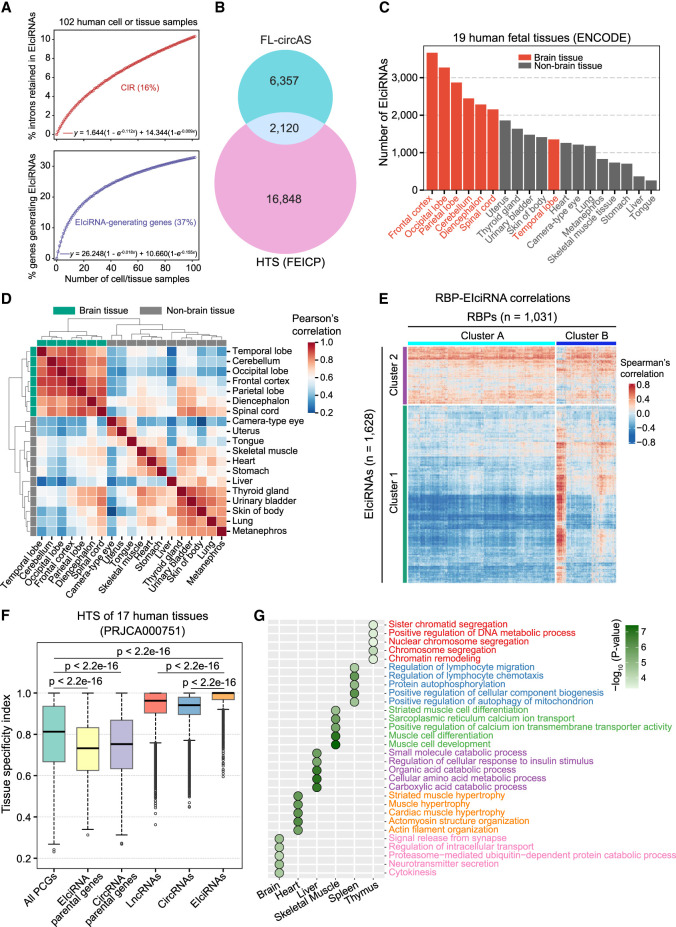
Identification and analyses of EIciRNAs in the human transcriptome. (*A*) The percentage of intron retained in circRNA (CIR) and genes generating EIciRNAs with the accumulating number of cell or tissue samples. The circles represent the mean values from 1000 iterations, and exponential curve fitting was applied. The corresponding equation and limit value are shown. (*B*) Overlap of EIciRNAs detected by FEICP and FL-circAS. (*C*) The number of EIciRNAs detected from ENCODE HTS data of 19 human fetal tissues. Twelve nonbrain tissues and seven brain tissues are labeled as gray and red, respectively. (*D*) Hierarchical clustering of EIciRNA BRPMs from 19 human fetal tissues. (*E*) Hierarchical clustering of Spearman's correlations of expression levels of RNA-binding proteins (RBPs) and EIciRNAs in 19 human fetal tissues. Cluster 1 and Cluster 2 are two row clusters for EIciRNAs, and Cluster A and Cluster B are two column clusters for RBPs. (*F*) Boxplots showing the tissue specificity index (tau) of PCGs, lncRNAs, circRNAs, and EIciRNAs, as well as parental genes of EIciRNAs and all circRNAs, from available HTS data sets (PRJCA000751) of 17 human tissues. *P*-values were calculated using the Wilcoxon rank-sum test. (PCGs) Protein-coding genes. (*G*) GO analysis of parental genes of EIciRNAs. The most significant five top GO terms are shown for six human tissues.

To systematically analyze EIciRNAs in humans, we collected 244 public paired-end RNA-seq data from 102 human cell lines and tissue types, and a total of 18,968 EIciRNAs from these data were identified with the FEICP pipeline, which overlapped with 2120 of the 8477 EIciRNAs identified by the FL-circAS database (Fig. 2A,B; Chiang et al. 2024). These samples represented ∼95% of the human PCGs, and the number of PCGs reached a saturation point (Supplemental Fig. S4A). By random sampling, we found that, overall, ∼16% of human introns contributed to the CIRs in EIciRNAs (Fig. 2A), and ∼37% of human PCGs could give rise to EIciRNAs (Fig. 2A). In addition, brain tissues and neural cells tended to have more EIciRNAs than did nonneural cells and tissues (Supplemental Fig. S4B). These data sets were generated by different laboratories and varied in many aspects, including sequencing library preparation, read length, sequencing depth, and data quality. Therefore, these data were not suitable for further in-depth analyses that require parallel comparisons. We then focused on the 38 ENCODE data sets, which were obtained from 19 human tissues with two biological replicates and had comparable and sufficient sequencing depths (373 million reads in depth on average). We found that both the number of EIciRNAs and the number of EIciRNA BSJs (normalized using backspliced reads per million [BRPM]) were generally greater in brain tissues than in nonbrain tissues (Fig. 2C; Supplemental Fig. S4C). Analysis of both the HTS and ONT data from eight human organs also showed that the brain and testis had more EIciRNAs than did the other tissues (Supplemental Fig. S4D). Seven brain tissues showed a distinct cluster of EIciRNAs, and the correlations of EIciRNA expression among brain tissues were greater than those among nonbrain tissues (Fig. 2D).

To analyze the potential roles of RBPs in the differential expression of EIciRNAs among tissues, we computed the Spearman's correlation coefficients of expression levels between all pairs of 1031 RBPs and 1628 EIciRNAs identified from the 19 tissues, followed by hierarchical clustering (Fig. 2E; Supplemental Methods). Two distinct RBP and EIciRNA clusters were revealed (Fig. 2E). A total of 1231 (75.6%) EIciRNAs formed cluster 1, which was negatively correlated with the expression of RBP cluster A (Fig. 2E). Cluster 2 had 397 (24.4%) EIciRNAs, and these genes were positively correlated with the expression of the RBPs in cluster A (Fig. 2E). Among the 1031 RBPs, binding profiles of 98 RBPs were characterized in the ENCODE eCLIP-seq data. Among them, 80 RBPs belong to cluster A and 18 belong to cluster B. We then calculated the binding density in both flanking introns, circularizing exons, and retained introns of EIciRNAs for each of the 98 RBPs (Supplemental Fig. S4E). We found that, compared with the RBPs in cluster B (positively regulated), those in cluster A (negatively regulated) had stronger binding density in both the circular exons and retained introns of these EIciRNAs, with no apparent difference in flanking introns (Supplemental Fig. S4E). These results suggested that RBPs complexly regulate the expression of EIciRNAs and that the majority of EIciRNAs are negatively correlated with RBPs. Protein–protein network analysis of the top 100 RBPs with the most negative correlations with EIciRNAs (Supplemental Methods) showed that they were involved in pathways such as RNA splicing, mRNA metabolic process, and post-transcriptional regulation of gene expression (Supplemental Fig. S4F). Several RBPs, including HNRNPK, SNRNP70, SRSF1, and SRSF2, were core nodes of this network (Supplemental Fig. S4F).

In one study, HTS data from 17 human tissues were obtained (Ji et al. 2019), and we analyzed the expression of PCGs, lncRNAs, and circRNAs (Fig. 2F). FEICP identified 4837 EIciRNAs from these tissues. Most of the EIciRNAs (3625, ∼75%) were expressed in only one tissue type, indicating that the EIciRNAs showed high tissue specificity (Supplemental Fig. S5A). We used the widely used tau method to quantify the tissue specificity of PCGs, lncRNAs, circRNAs, EIciRNAs, and the parental genes of EIciRNAs and circRNAs (Supplemental Methods) (Fig. 2F; Yanai et al. 2005). Compared with those of all PCGs, the other three types of RNAs showed greater tissue specificity, among which EIciRNAs showed the strongest tissue specificity (Fig. 2F). In contrast, we also found that the expression of parental genes of both EIciRNAs and circRNAs showed lower tissue specificity than did those of the all PCGs control (Fig. 2F). We then performed a gene enrichment analysis of those genes, whose mRNAs were expressed without strict tissue specificity but generated EIciRNAs in only one tissue type (Fig. 2G). We found that these parental genes were enriched in GO terms highly correlated with tissue identity (Fig. 2G). We further analyzed 70 ENCODE RNA-seq data sets from 18 tissues of four adults (Supplemental Fig. S5B). At least 50 EIciRNAs detected eight tissues from all individuals, after which hierarchical clustering of correlations between all pairs of these tissues was performed based on the expression of the EIciRNAs (Supplemental Fig. S5B). Biologically similar tissues were clustered together, which showed that EIciRNAs could be applied as markers of tissue identity (Supplemental Fig. S5B).

### Genes generating EIciRNAs are more actively transcribed

**Figure 3.**
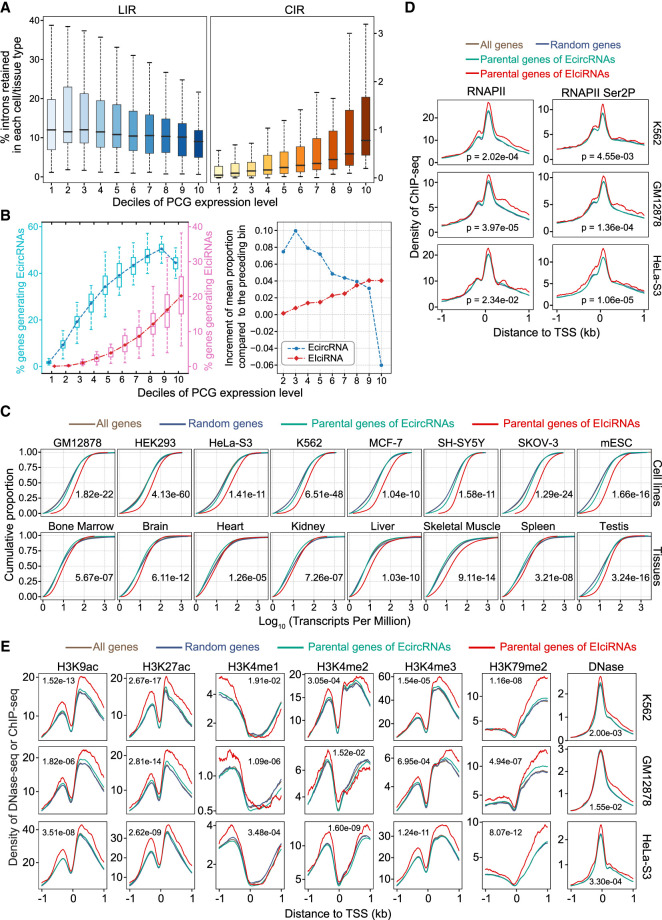
Parental genes of EIciRNAs are actively transcribed. (*A*) Boxplots displaying the distributions of percentages of CIRs and introns retained in linear RNA (LIRs) in 10 groups (deciles) with ranked expression levels. (*B*) Boxplots displaying the distributions of percentage of genes generating EcircRNAs or EIciRNAs in 10 groups (deciles) with ranked expression levels. (*Left*) The mean proportion in each group is indicated as a point. (*Right*) The increment of mean proportion of genes generating EcircRNAs or EIciRNAs compared with the preceding bin is represented as a point for each bin. (*C*) Cumulative distribution of expression levels (transcripts per million [TPM]) of all genes, random genes, parental genes of EcircRNAs, and EIciRNAs in eight cell lines and eight tissues. (All genes) All expressed PCGs with TPM ≥ 1, (random genes) 2000 genes randomly selected from all genes, (parental genes of EIciRNAs) PCGs generating EIciRNAs, and (parental genes of EcircRNAs) PCGs generating EcircRNAs but no EIciRNAs. (*D*) Distributions of RNAPII and RNAPII Ser2P ChIP-seq signals around the transcription start site (TSS) regions of the indicated groups of genes in the K562, GM12878, and HeLa-S3 cell lines. (*E*) DNase-seq and ChIP-seq signals of H3K9ac, H3K27ac, H3K4me1, H3K4me2, H3K4me3, and H3K79me2 around the TSS regions of the indicated group of genes in the K562, GM12878, and HeLa-S3 cell lines. In *C*–*E*, *P*-values were calculated with the Wilcoxon rank-sum test to compare the parental genes of EIciRNAs and parental genes of EcircRNAs.

Next, we examined the gene expression features of the EIciRNA-generating genes. We detected the CIRs and quantified gene expression from the 38 RNA-seq data sets used in Figure 2C. For comparison, we analyzed genes with LIRs using 71 poly(A)-plus RNA-seq data from 30 human tissues from ENCODE. All the expressed PCGs (TPM ≥ 1) were divided into 10 bins according to their TPM, and the fraction of LIRs or CIRs was calculated for each bin (Fig. 3A). The overall proportion of CIRs increased with increasing parental gene expression (Fig. 3A). However, the overall proportion of LIRs tended to negatively correlate with parental gene expression (Fig. 3A), and this feature of LIRs was reported in a previous study (Braunschweig et al. 2014). We compared the proportions of genes producing EIciRNAs or EcircRNAs in different expression-level bins and found that the proportions of genes generating either EIciRNAs or EcircRNAs generally increased with increasing gene expression levels (Fig. 3B). However, there was a notable decline for the proportion of EcircRNA-producing genes in the highest expression-level bin (Fig. 3B). To quantify the increasing rate for proportions of genes generating EcircRNAs or EIciRNAs, we calculated mean values of the proportions for each bin and then obtained their increment compared with the preceding bin (Fig. 3B). It was found that with the increase of gene expression levels, the accelerating trend increased gradually for genes generating EIciRNAs, whereas the trend decreased from the second bin on for genes generating EcircRNAs (Fig. 3B). To investigate the expression levels of genes that give rise to EIciRNAs versus EcircRNAs, we analyzed RNA-seq data from 12 cell lines and 17 tissues, whose sample preparation allowed analyses of EIciRNAs and EcircRNAs together (Supplemental Methods) (Fig. 3C; Supplemental Fig. S6A). In all these cell lines and tissues, the expression levels of EIciRNA-generating genes were significantly higher than those of genes generating only EcircRNAs but not EIciRNAs (Fig. 3C; Supplemental Fig. S6A). The expression levels of EIciRNA-generating genes were also greater than those of either all expressed PCGs (overall-control) or 2000 randomly selected PCGs (random-control) (Fig. 3C; Supplemental Fig. S6A).

To investigate whether the relatively higher expression levels of EIciRNA-generating genes were associated with more active transcription, we assessed the RNAPII and RNAPII Ser2P signals on the corresponding genes in the K562, GM12878, and HeLa-S3 cell lines (Fig. 3D). These three cell lines were the only ones with chromatin-immunoprecipitation sequencing (ChIP-seq) data for both RNAPII and RNAPII Ser2P that were available in The ENCODE Project. We found that in these three cell lines, the ChIP-seq signals of both RNAPII and RNAPII Ser2P around the transcription start site (TSS) of EIciRNA-generating genes were significantly greater than those of genes generating only EcircRNAs but not EIciRNAs and were significantly greater than those of the overall-control and random-control genes (Fig. 3D). We also analyzed six cell lines for which only RNAPII ChIP-seq data were available (Supplemental Fig. S6B). We found that in these cell lines, the ChIP-seq signals of RNAPII at the TSSs of EIciRNA-generating genes were significantly greater than those of genes generating only EcircRNAs but no EIciRNAs and were greater than those of the overall-control and random-control groups (Supplemental Fig. S6B). We further assessed nascent RNAs in four cell lines using public transient transcriptome sequencing of nascent transcripts (TT-seq) data (Supplemental Fig. S6C). In these four cell lines, EIciRNA-generating genes were more actively transcribed than were the other genes (Supplemental Fig. S6C).

Six histone modifications (H3K9ac, H3K27ac, H3K4me1, H3K4me2, H3K4me3, and H3K79me2) and chromatin accessibility associated with active transcription were subsequently evaluated in K562, GM12878, and HeLa-S3 cells (Fig. 3E). We observed that, in all three cell lines, the DNase-seq and ChIP-seq signals of all six histone modifications were significantly greater around the TSSs of EIciRNA-generating genes than around those generating only EcircRNAs but not EIciRNAs and were also greater than those of the overall-control and random-control groups (Fig. 3E). However, the difference was weaker, or not significant, or not consistent among cell types for four histone modifications (H3K9me3, H3K27me3, H3K36me3, and H4K20me1) associated with repressed transcription (Supplemental Fig. S6D). To further investigate the correlation between the generation of EIciRNAs and the levels of these epigenetic marks, we compared the EIciRNA-generating genes with genes not producing EIciRNAs but having expression levels comparable to those of EIciRNA-generating genes (EIciRNA-match genes) (Supplemental Fig. S7A). We found that the signals around the TSSs of EIciRNA-generating genes were greater than those of EIciRNA-match genes for most activation marks (Supplemental Fig. S7B). When the top 10% of genes with high expression levels were examined as two groups with or without EIciRNA-generating, the signals around the TSSs of EIciRNA-generating genes were again generally higher than those of without EIciRNA-generating genes for most activation marks (Supplemental Fig. S7B). Overall, the genes generating EIciRNAs tended to be associated with active transcription and the corresponding epigenetic marks.

### CIRs are distinct from LIRs and other introns

**Figure 4.**
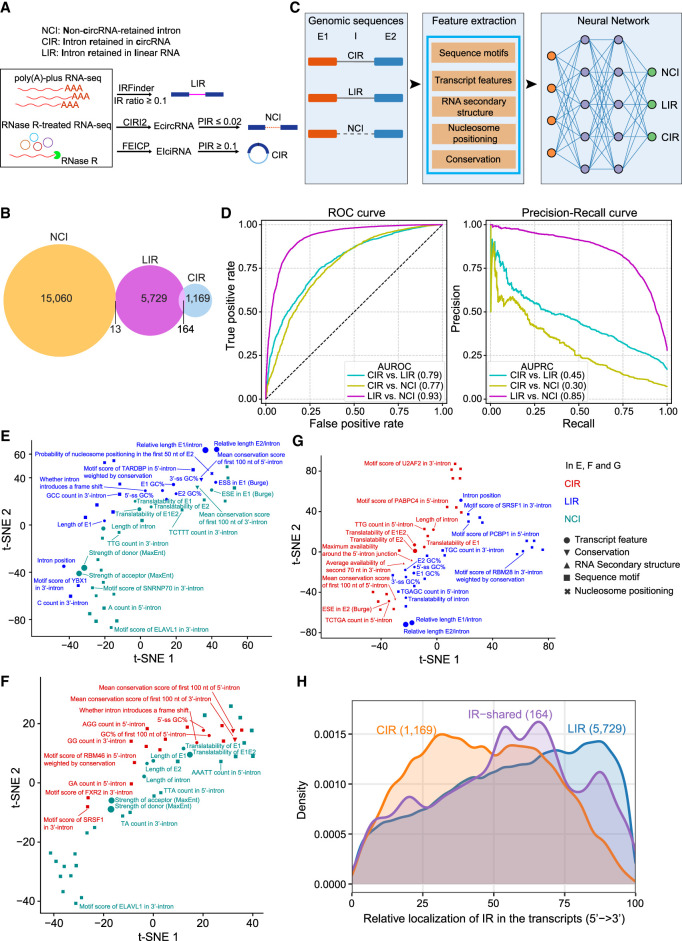
Features of CIR compared with LIR and the other introns. (*A*) A schematic demonstration of the identification of LIR, NCI, and CIR. LIR was detected from poly(A)-plus RNA-seq data through IRFinder with the cutoff (IRratio ≥ 0.1). CIR was detected from RNase R–treated RNA-seq data using FEICP with the cutoff (percent intron retention [PIR] ≥ 0.1). NCI represents spliced introns of EcircRNAs with the cutoff (PIR ≤ 0.02). (*B*) Venn diagram showing the overlap of LIR, NCI, and CIR in HEK293 cells. (*C*) Illustrative diagram for training the neural network (NNetwork) to distinguish LIR, NCI, and CIR. (*D*) Performance of the NNetwork in distinguishing three groups of introns from each other. ROC curves and precision-recall curves were plotted for pairwise comparisons, with the corresponding area under the ROC curve (AUROC) and area under the precision-recall curve (AUPRC) values shown in parentheses. (*E–G*) Scatter plots showing the results of t-SNE analysis of the top 50 genomic features for distinguishing NCI and LIR (*E*), NCI and CIR (*F*), and LIR and CIR (*G*). The colors indicate the group of introns with which a feature was positively correlated in the pairwise comparison. The point size indicates the feature importance. (*H*) Density curves showing the genomic distribution of LIR, CIR, and overlapped IR across their host transcripts.

We wondered whether CIRs had some specific sequence features, and we analyzed three groups of introns, namely, the CIRs, LIRs, and non-circRNA-retained introns (NCIs), in HEK293 cells (Supplemental Methods) (Fig. 4A; Supplemental Tables S1, S3). NCIs refer to introns located between circularized exons in the genome but are not retained during backsplicing to form EcircRNAs. The NCIs and LIRs had little overlap, and a small portion of the CIRs overlapped with the LIRs (164 of 1333 CIRs, ∼12.30%) (Fig. 4B). Similar results were found by analyzing published RNA-seq data from four cell lines, namely, HEK293, HeLa, K562, and SH-SY5Y cells (Supplemental Fig. S8A). Therefore, CIRs were generally distinct from LIRs, and the sequence features of CIRs were subjected to further investigations.

We analyzed the introns belonging to only one group (15,060 NCIs, 5729 LIRs, and 1169 CIRs) and extracted 1309 sequence features of these introns and the corresponding upstream exons (E1) and downstream exons (E2) (Fig. 4C; Supplemental Table S4). These features included five aspects, namely, transcript features, sequence conservation, RNA secondary structure, sequence motifs, and nucleosome positioning (Supplemental Methods) (Fig. 4C; Supplemental Table S4). All these features were subsequently used to train a three-layer neural network (NNetwork) (LeCun et al. 2015; Yeom et al. 2021), which aimed to predict whether an intron belonged to the NCI, CIR, or LIR (Fig. 4C; Supplemental Methods). We then evaluated the performance of this NNetwork model using receiver operating characteristic (ROC) curves and precision-recall curves (Fig. 4D). By computing the area under the ROC curve (AUROC) and the area under the precision-recall curve (AUPRC), we found that the NNetwork model showed the highest distinction between the NCIs and LIRs (AUROC = 0.93, AUPRC = 0.85), and the CIRs could also be distinguished from the NCIs and LIRs (AUROC = 0.77 and 0.79, AUPRC = 0.30 and 0.45, respectively) (Fig. 4D). These results showed that, for these sequence features, the NCIs and LIRs were the most different, whereas the CIRs and NCIs or the CIRs and LIRs were also significantly different, although to a lesser extent. We also extracted the 1309 genomic sequence features of introns for linear RNAs, which were used to predict whether an intron was retained by training a three-layer NNetwork (NNetwork-linear), which achieved an AUROC of 0.89 and an AUPRC of 0.37 (Supplemental Fig. S8B). When applying this two-classifier based on LIRs to distinguish CIRs from NCIs, we observed a noticeable decrease in both the AUROC (0.72) and AUPRC (0.27) compared with those of the three-class classifier (Supplemental Fig. S8C). We thus confirmed that CIRs were inherently different from LIRs.

We next assessed the importance of each of the 1309 features in distinguishing groups of introns from each other. The top 50 features distinguishing the two groups were extracted and further analyzed (Fig. 4E–G; Supplemental Table S4). Consistent with the findings of previous reports (Braunschweig et al. 2014; Yeom et al. 2021), a lower SS strength, higher intron GC content, and shorter introns were highly predictive of LIRs over NCIs and of CIRs over NCIs (Fig. 4E,F; Supplemental Fig. S9A). We compared the SS strength of the top 500 CIRs or LIRs with that of the corresponding bottom 500 IRs according to their percent intron retention (PIR) (Supplemental Fig. S9B). We found that, for LIRs, the top 500 had lower 5′ and 3′ SS strengths than did the bottom 500 (Supplemental Fig. S9B), whereas for CIRs, the top 500 had lower 5′ SS strength than did the bottom 500, and there was no significant difference in the 3′ SS strength (Supplemental Fig. S9B).

CIRs had higher sequence conservation scores at both the 5′- and 3′-ends of the introns compared with NCIs, which might imply the conserved regulatory functions of CIRs (Fig. 4F). The motif scores of several RBPs, including SRSF1, FXR2, and ELAVL1, had either positive or negative correlations with the CIRs. (Fig. 4F). We further integrated the eCLIP-seq data of 134 RBPs from ENCODE and compared their binding in CIRs over NCIs (Supplemental Methods) (Supplemental Fig. S9C; Van Nostrand et al. 2020). Eighty-nine of the 134 RBPs had significantly more binding sites in the CIR group than in the NCI group (Supplemental Fig. S9C). Additionally, among the 50 features predictive of CIRs over NCIs, two were for exons that flanked the retained introns in EIciRNAs; these exons were shorter in length and had lower translatability (Fig. 4F).

Several features, including a lower GC content, a longer length, a greater propensity to form a local secondary structure, a greater sequence conservation of the 5′-end of the intron, and a smaller position (toward the 5′-end of the gene) of the intron, were predictive of CIRs over LIRs (Fig. 4G; Supplemental Fig. S9A). By plotting the retained introns along their host transcripts, we observed an evident trend that CIRs peaked at the 5′ portion of the transcripts, whereas LIRs peaked at the 3′ portion of the transcripts in five data sets from four cell lines (Fig. 4H; Supplemental Fig. S9D). Consistently, the 164 introns shared by CIRs and LIRs in our HEK293 data set peaked in the middle of the transcripts (Fig. 4H). We also plotted the relative positions of the start exons and end exons of the EIciRNAs along the transcripts and found that the start exons of the EIciRNAs peaked at the 5′ region of the transcripts, whereas the end exons showed a relatively uniform distribution across the middle of the transcripts (Supplemental Fig. S9E). This finding was consistent with the reported localization pattern of start and end exons for circRNAs (Guo et al. 2014; Westholm et al. 2014; Rybak-Wolf et al. 2015). LIRs tend to locate to the 3′ region of genes, and a position closer to the 3′-end might be associated with the functional role of LIRs in regulating mRNA translation and decay (Braunschweig et al. 2014; Mauger et al. 2016; Schmitz et al. 2017; Wong and Schmitz 2022).

### A genome-wide CRISPR-Cas9 screen for genes regulating CIRs identifies SRSF1

**Figure 5.**
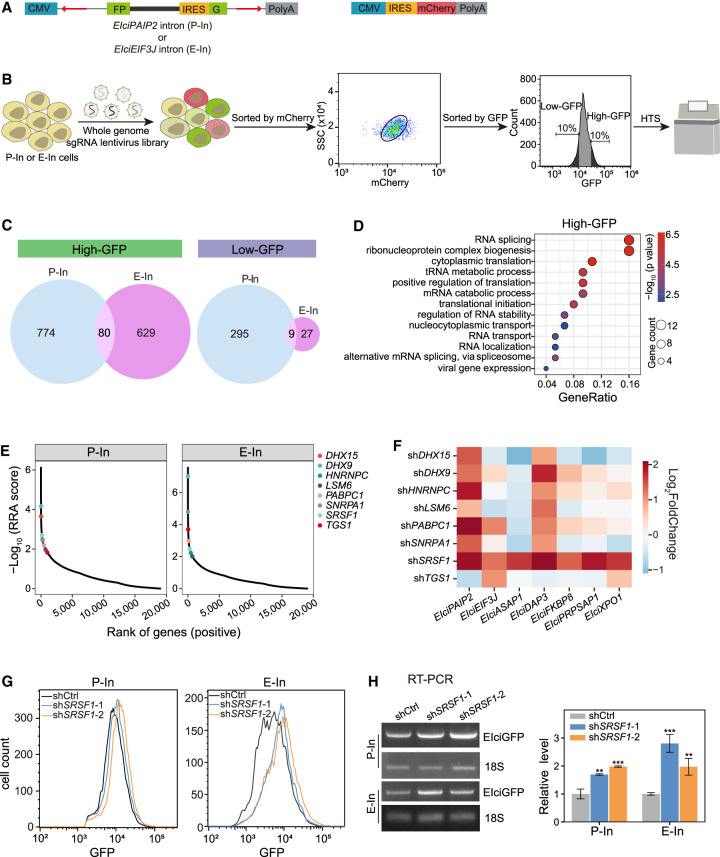
Genome-wide CRISPR screen identifies regulators of EIciRNA biogenesis. (*A*) Constructs for expressing GFP and mCherry protein reporters. The reverse complementary sequence (red arrows) in the flanking introns was used for the circularization of split GFP. The retained introns of two reported EIciRNAs (*EIciPAIP2* and *EIciEIF3J*) were inserted between the split GFP (FP) and IRES sequences. Two GFP reporters (P-In for *EIciPAIP2* and E-In for *EIciEIF3J*) were stably integrated into the genome of HEK293 cells along with the mCherry construct (Supplemental Methods). (*B*) Procedure of genome-wide CRISPR screen. Two reporter cells (P-In or E-In) were infected with the lentivirus sgRNA library and selected with 1 μg/mL puromycin for 7 d. The infected cells expressing mCherry proteins were gated with FACS, and 10% cell populations of high-GFP and low-GFP were used for genomic extraction, PCR amplification, and HTS. (*C*) Venn diagram showing the overlapped genes with significantly enriched sgRNAs in the screens of P-In or E-In reporters. (*D*) GO analysis of 80 genes overlapped in high-GFP of both P-In and E-In groups. (*E*) Ranks of eight RBPs enriched in the biological process of RNA splicing. Ranks were calculated using MAGeCK (Li et al. 2014). (*F*) Heatmap showing the relative fold change of seven EIciRNAs examined by RT-qPCR when eight RBPs were individually knocked down by shRNA. (*G*) Flow cytometric analysis showing the GFP expression in P-In (*left*) and E-In (*right*) cells upon *SRSF1* knockdown. (shCtrl) shRNA with scrambled sequences, (sh*SRSF1*-1, sh*SRSF1*-2) two independent shRNAs against *SRSF1*. (*H*) Semiquantitative RT-PCR gels and quantification of the EIciGFP levels in P-In and E-In cells after *SRSF1* knockdown. 18S rRNA was used as the loading control. Error bars represent standard deviation (SD) in triplicate experiments. *P*-values from two-tailed Student's *t*-test. (**) *P* < 0.01, (***) *P* < 0.001.

The introns retained in CIR were unique, and we subsequently sought to identify regulators of IR in EIciRNAs by performing a genome-wide CRISPR-Cas9 screening. To this end, we constructed an intron-split-GFP circRNA reporter construct (EIciGFP) in which the intron retained in *EIciEIF3J* or *EIciPAIP2* was inserted between the split GFP sequence and the internal ribosome entry site (IRES) (Fig. 5A; Yang et al. 2017). EIciGFP could be produced when backsplicing occurred and the intron was retained, and it was found to localize in the nucleus (Fig. 5A; Supplemental Fig. S10A). When the intron was not retained, EcircGFP was produced; EcircGFP was localized mainly in the cytoplasm to serve as a translational template for the GFP (Fig. 5A; Supplemental Fig. S10A). As a control, we constructed an mCherry reporter, which was composed of the same promoter and used IRES as the EIciGFP reporter (Fig. 5A). The EIciGFP reporter with the intron retained in *EIciEIF3J* or *EIciPAIP2* and the mCherry reporter were used to construct a stable HEK293 cell line, which was termed E-In cells or P-In cells (Fig. 5A; Supplemental Fig. S10A–E).

A whole-genome CRISPR-Cas9 knockout lentivirus library (Brunello), which was composed of 76,441 single-stranded guide RNAs (sgRNAs) targeting 19,114 genes in the human genome (Doench et al. 2016), was transfected into either E-In cells or P-In cells (Fig. 5B). Using fluorescence-activated cell sorting (FACS), we isolated cells that had the same mCherry fluorescence intensity as nonlentivirus-infected cells and collected the top 10% and the bottom 10% of the cells according to GFP expression (high-GFP or low-GFP group) (Fig. 5B). sgRNAs obtained from the input (bulk cells without sorting), high-GFP, and low-GFP groups were subjected to HTS (Fig. 5B; Supplemental Table S5).

Genes targeted by sgRNAs that were significantly enriched in high-GFP or low-GFP group compared with the input group were identified. From the high-GFP group, 854 and 709 candidate genes were identified from P-In and E-In cells, respectively (Fig. 5C), which had an overlap of 80 genes (Fig. 5C). For the low-GFP group, we obtained 304 and 36 candidate genes in P-In and E-In, respectively, which had an overlap of nine genes (Fig. 5C). In this study, we focused on 80 genes, whose expression was knocked out by CRISPR-Cas9, resulting in GFP expression. GO analysis of these 80 genes revealed enrichment in biological processes related to RNA metabolism and gene expression, such as RNA splicing and ribonucleoprotein complex biogenesis (Fig. 5D). Among these genes, 26 were known RBP genes (Gerstberger et al. 2014), and eight of them encoded factors associated with RNA splicing (Fig. 5E; Supplemental Fig. S10F). All these eight RBPs were also detected to be negatively correlated with EIciRNA expression (Fig. 2E; Supplemental Fig. S10G). We then examined these eight RBPs experimentally by knocking down with the corresponding shRNAs and investigated the expression levels of seven EIciRNAs, including *EIciPAIP2* and *EIciEIF3J* (Fig. 5F). *SRSF1* was the only gene whose knockdown led to significantly increased levels of all seven EIciRNAs (Fig. 5F). Knockdown of *SRSF1* with two independent shRNAs in the reporter cell lines P-In and E-In resulted in increased EIciGFP and GFP levels (Fig. 5G,H; Supplemental Fig. S10H,I). Therefore, SRSF1 was identified as a candidate of negative regulators for CIRs through genome-wide CRISPR-Cas9 screening with two EIciRNA reporters.

### SRSF1 regulates EIciRNA biogenesis

**Figure 6.**
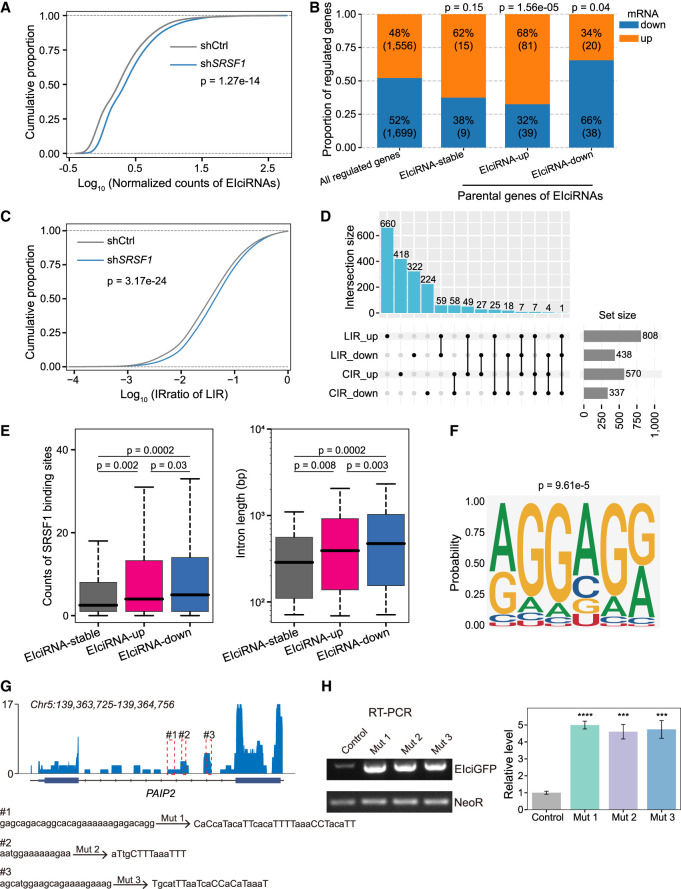
SRSF1 binds to CIR to inhibit EIciRNA biogenesis. (*A*) Cumulative distribution of normalized EIciRNA expressions in HEK293 cells treated with shRNA targeting *SRSF1*. (shCtrl) shRNA with scrambled sequences, (sh*SRSF1*) shRNA against *SRSF1*. (*B*) Changes of EIciRNA parental genes upon *SRSF1* knockdown. (EIciRNA-up) Up-regulated EIciRNAs upon *SRSF1* knockdown, (EIciRNA-down) down-regulated EIciRNAs upon *SRSF1* knockdown, and (EIciRNA-stable) unaltered EIciRNAs upon *SRSF1* knockdown. (*C*) Cumulative distribution of the IRratio of LIR in HEK293 cells treated with shRNA targeting scramble sequence or *SRSF1*. (*D*) UpSet plot showing the overlap of genes harboring LIR or CIR regulated by SRSF1. (*E*) Boxplots displaying the counts of SRSF1 binding sites in CIR and the length of CIR in the indicated groups. (*F*) Predicted binding motif from SRSF1 iCLIP-seq reads mapped to introns retained in up-regulated EIciRNAs upon *SRSF1* knockdown. (*G*) Integrative Genomics Viewer (IGV) (Robinson et al. 2011) snapshot showing the SRSF1 iCLIP-seq signals in the *EIciPAIP2* locus. Three SRSF1 binding GA-rich regions in the retained intron of *EIciPAIP2* are framed as red dotted lines and labeled as #1, #2, and #3, respectively. (*H*) Semiquantitative RT-PCR gels and the quantification of EIciGFP levels in HEK293 cells after transfection of indicated mutation plasmids. The eukaryotic resistance gene (NeoR) of the plasmids was used as the loading control. In *A* and *C*, *P*-values were calculated with the Wilcoxon rank-sum test. In *B*, *P*-values calculated with the chi-squared test were shown for the comparison of proportion between the indicated group of genes against all regulated genes by *SRSF1* knockdown. In *E*, *P*-values were calculated with the two-tailed Student's *t*-test. In *H*, error bars represent SD in triplicate experiments, and *P*-values were calculated with the two-tailed Student's *t*-test. (***) *P* < 0.001, (****) *P* < 0.0001.

To further investigate the roles of SRSF1 in CIRs and EIciRNAs, we generated a stable *SRSF1*-knockdown (sh*SRSF1*) HEK293 cell line (Supplemental Fig. S11A). Both poly(A)-plus and RNase R–treated RNA-seq were performed, after which the expression levels of mRNAs and EIciRNAs were quantified in the control and sh*SRSF1* cells (Fig. 6A,B; Supplemental Tables S6, S7).

The global expression levels of EIciRNAs significantly increased upon *SRSF1* knockdown (Fig. 6A; Supplemental Table S6). In detail, 56% (658) of the EIciRNAs were up-regulated (EIciRNA-up), 33% (382) of the EIciRNAs were down-regulated (EIciRNA-down), and 11% (127) of the EIciRNAs were not significantly changed (EIciRNA-stable) (Supplemental Fig. S11B). When the expression of EIciRNA parental genes was examined, we found that, compared with those of EIciRNA-stable parental genes, significantly more EIciRNA-up parental genes had increased mRNA levels, whereas significantly more EIciRNA-down parental genes had decreased mRNA levels (Fig. 6B; Supplemental Table S7). By performing global run-on sequencing (GRO-seq) upon *SRSF1* knockdown, we found that the global levels of nascent RNAs from all PCGs expressed in HEK293 cells were decreased (Supplemental Fig. S11C). Those EIciRNA-stable parental genes showed no significant difference to all PCGs in the nascent RNA levels (Supplemental Fig. S11C). EIciRNA-down parental genes showed significantly larger decreases, whereas EIciRNA-up parental genes had significantly fewer decreases in nascent RNA levels, compared with all PCGs (Supplemental Fig. S11C). It seemed that the overall effect of *SRSF1* knockdown would lead to down-regulation in the transcription, whereas EIciRNA-up parental genes had a tendency to resist the down-regulation. Overexpression of FLAG-tagged *SRSF1* resulted in a significant decrease in the nascent production of EIciRNAs and the corresponding mRNAs of their parental genes in the three EIciRNA/mRNA pairs examined (Supplemental Fig. S11D,E).

We then investigated the alterations in LIRs and CIRs upon the *SRSF1* knockdown. We found that the IR ratio of LIRs significantly increased upon *SRSF1* knockdown (Fig. 6C; Supplemental Table S8), consistent with the findings of previous reports in HepG2 cells (Middleton et al. 2017). The majority (1741/1879, ∼93%) of genes with significant changes in IR showed only regulated LIRs or regulated CIRs (Fig. 6D). Although most of the genes generally possessed multiple introns, the IR of most of the genes was unidirectionally regulated by SRSF1, with LIRs in 982/1041 (∼94%) genes being either up-regulated or down-regulated and CIRs in 642/700 (∼92%) genes being either up-regulated or down-regulated (Fig. 6D).

Reanalysis of published SRSF1 iCLIP-seq data from HKE293 cells revealed that there were more SRSF1 binding sites in the retained introns of EIciRNAs regulated than in those unregulated by SRSF1 (Fig. 6E; Howard et al. 2018). Moreover, there was no significant difference in SRSF1 binding to exons or flanking introns of EIciRNAs (Supplemental Fig. S11F). The length of introns was significantly longer in EIciRNAs regulated by SRSF1 than in those unregulated by SRSF1 (Fig. 6E). Compared with introns retained in up-regulated EIciRNAs, those in down-regulated EIciRNAs were longer and had more SRSF1 binding sites (Fig. 6E). When the number of SRSF1 binding sites was normalized to the intron length, there was no significant difference in the density of SRSF1 binding sites among the introns retained in the stable, up-regulated, or down-regulated EIciRNAs (Supplemental Fig. S11G). Consistent with these findings, the metaprofiles of SRSF1 binding to the three groups of EIciRNAs were not significantly different (Supplemental Fig. S11H). Therefore, we speculated that the complicated changes in CIRs resulting from *SRSF1* knockdown were more related to the length and might also be regulated in combination with some other RBPs required further investigation.

Analysis of SRSF1 iCLIP-seq reads mapped to retained introns in the up-regulated EIciRNAs identified a purine-rich motif (Fig. 6F). We found via iCLIP-seq that introns of *EIciPAIP2* and *EIciEIF3J* harbored several SRSF1 binding sites, and among them, three and two were found to be purine-rich in introns of *EIciPAIP2* and *EIciEIF3J*, respectively (Fig. 6G; Supplemental Fig. S11I). We then experimentally examined the SRSF1 binding sites in introns of *EIciEIF3J* and *EIciPAIP2* (Fig. 6G; Supplemental Fig. S11I). Random mutation of purines to pyrimidines in these sequences with EIciGFP reporters led to significantly increased levels of the corresponding EIciGFP (Fig. 6G,H; Supplemental Fig. S11I,J). Overall, we concluded that SRSF1 could suppress CIR and thus the biogenesis of a portion of EIciRNAs by binding to specific motifs in introns of parental genes, although the overall regulatory mechanisms of EIciRNA biogenesis are complicated.

### Regulation of CIRs and EIciRNAs has functional roles in neuronal differentiation

**Figure 7.**
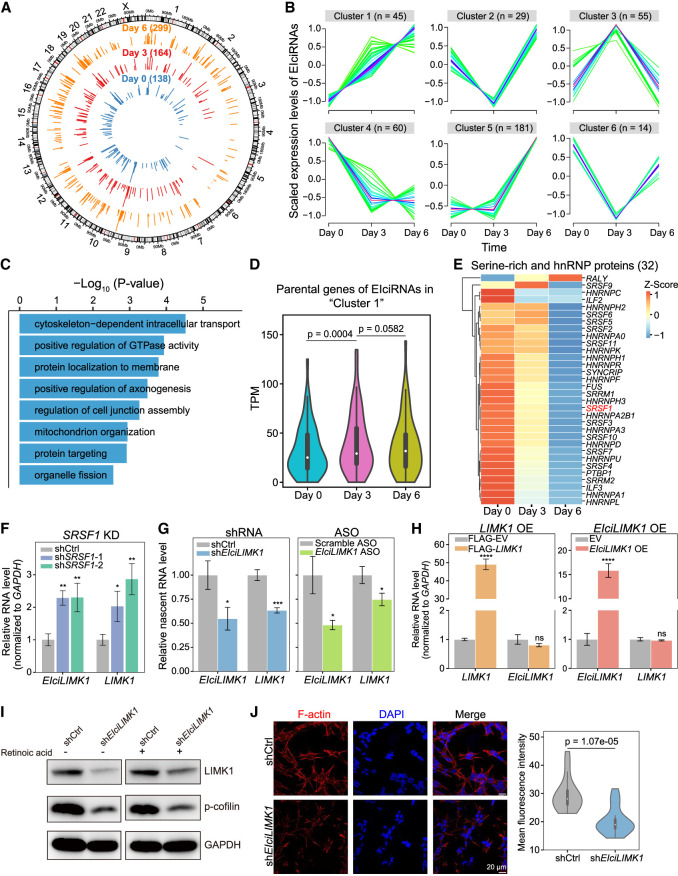
Functional roles of EIciRNAs in neuronal differentiation. (*A*) Circos plots (Krzywinski et al. 2009) showing genomic distribution (hg38) and backspliced reads per million (BRPM) of EIciRNAs at day 0, day 3, or day 6 after retinoic acid (RA)–induced SH-SY5Y differentiation. Two biological replicates were used for analysis. (*B*) Fuzzy clustering showing the temporal expression patterns of EIciRNAs during SH-SY5Y cell differentiation. (*C*) Enriched GO terms of parental genes of EIciRNAs (cluster 1 in *B*). (*D*) Violin plots displaying the expression levels (TPM) of EIciRNA (cluster 1) parental genes during SH-SY5Y cell differentiation. (*E*) Heatmap showing the expression levels of 32 serine-rich (SR) and HNRNP proteins during SH-SY5Y cell differentiation (Papasaikas et al. 2015). (*F*) RT-qPCR analysis of *EIciLIMK1* and *LIMK1* mRNA expression in SH-SY5Y cells after *SRSF1* knockdown. (shCtrl) shRNA with scrambled sequences, (sh*SRSF1*-1 and sh*SRSF1*-2) two independent shRNAs against *SRSF1*. (*G*) Nuclear run-on assay showing the nascent level of *LIMK1* mRNA in SH-SY5Y cells after *EIciLIMK1* knockdown with shRNA or ASO against the *EIciLIMK1* BSJ. (Scramble ASO) ASO with scrambled sequences. (*H*) RT-qPCR analysis of *EIciLIMK1* and *LIMK1* mRNA expression in SH-SY5Y cells after overexpression of *LIMK1* (*left*) and *EIciLIMK1* (*right*). (*I*) Western blot showing the expression levels of LIMK1 and phosphorylated cofilin (p-cofilin) protein levels in SH-SY5Y cells in uninduced and RA-induced SH-SY5Y cells after *EIciLIMK1* knockdown. GAPDH was used as the loading control. (*J*) Representative immunofluorescence (IF) images of F-actin in RA-induced SH-SY5Y cells after *EIciLIMK1* knockdown (*left*). The F-actin fluorescence intensity was quantified with Fiji (*right*). N = 40. In *D* and *J*, *P*-values were calculated with the Wilcoxon rank-sum test. In *F*–*H*, error bars represent SD in triplicate experiments, and *P*-values were calculated with the two-tailed Student's *t*-test. (*) *P* < 0.05, (**) *P* < 0.01, (***) *P* < 0.001, (****) *P* < 0.0001.

To investigate the biological relevance of CIR and EIciRNAs, we applied an established neuronal differentiation model by inducing SH-SY5Y cells with retinoic acid (RA) (Supplemental Fig. S12A–C; Ross et al. 1983; Rybak-Wolf et al. 2015). HTS of RNAs from noninduced and RA-induced cells was performed to evaluate circRNAs and mRNAs, each with two biological replicates (Supplemental Table S9). We detected 384 high-confidence EIciRNAs in total (Supplemental Fig. S12D; Supplemental Table S9), among which 138, 164, and 299 EIciRNAs were identified on days 0 (noninduced), 3, and 6 of SH-SY5Y differentiation, respectively (Fig. 7A). Temporal clustering of these EIciRNAs revealed six clusters (Fig. 7B). To investigate the EIciRNAs that could promote SH-SY5Y differentiation, we focused on cluster 1, in which most EIciRNAs increased continuously during differentiation (Fig. 7B). Gene enrichment analysis revealed that the parental genes of these EIciRNAs were enriched in pathways associated with neurological activities and cell polarity (Fig. 7C). Moreover, the overall expression levels of the parental genes of these EIciRNAs increased significantly from day 0 to day 3, whereas the increase was not significant from day 3 to day 6 (Fig. 7D), implying that these EIciRNAs might play regulatory roles in the early stages of SH-SY5Y differentiation. When the expression of the 141 well-defined splicing factors was analyzed (Papasaikas et al. 2015), we found that their overall expression levels decreased during differentiation (Supplemental Fig. S12E,F).

Specifically, among the 32 serine-rich and HNRNP splicing factors that are known to regulate splicing by direct intronic binding, 27 showed a decreasing trend during differentiation (Fig. 7E). The opposite trend in expression between the levels of these splicing factors and EIciRNAs was consistent with the finding that the expression levels of the majority of RBPs were negatively correlated with those of EIciRNAs (Fig. 2E). We noticed that the serine/threonine kinase *LIMK1*, which was enriched in the GO terms “positive regulation of GTPase activity” and “positive regulation of axonogenesis,” could generate an EIciRNA (*EIciLIMK1*) in cluster 1 (Supplemental Fig. S13A). Considering that SRSF1 has previously been shown to play a role in regulating EIciRNA biogenesis (Figs. 2E, 4F, 5, 6; Supplemental Figs. S4F, S9C), we experimentally examined whether SRSF1 regulated *EIciLIMK1* in SH-SY5Y cells (Fig. 7F). *SRSF1* knockdown significantly increased in the expression of the *EIciLIMK1* and *LIMK1* mRNA (Fig. 7F; Supplemental Fig. S13B). RNA immunoprecipitation (RIP) experiments showed that SRSF1 bound to the flanking introns, the circular exons, and the retained intron of the *EIciLIMK1* locus (Supplemental Fig. S13C,D).

To determine whether *EIciLIMK1* plays a role in neuronal differentiation, we knocked down *EIciLIMK1* with shRNAs or RNase H–based antisense oligonucleotides (ASOs) (Supplemental Fig. S13E) and found that the expression level of *LIMK1* mRNA significantly decreased upon *EIciLIMK1* knockdown (Supplemental Fig. S13E). Further, nuclear run-on assays showed that knockdown of *EIciLIMK1* resulted in significantly decreased *LIMK1* transcription levels (Fig. 7G). However, the expression level of *EIciLIMK1* did not change upon *LIMK1* mRNA knockdown or overexpression (Supplemental Fig. S13F; Fig. 7H). The mRNA levels of *LIMK1* were unaffected by overexpression of *EIciLIMK1* with plasmid (Fig. 7H). These results indicated that *EIciLIMK1* could promote the transcription of the *LIMK1* gene in *cis*.

Western blot analysis revealed that the levels of both LIMK1 protein and Ser-3-phosphorylation of cofilin (p-cofilin) significantly decreased after knockdown of either *LIMK1* mRNA or *EIciLIMK1* in SH-SY5Y cells (Supplemental Fig. S13G,H; Fig. 7I). Cofilin, a substrate of LIMK1, can be phosphorylated to p-cofilin, which is involved in the formation of F-actin during neuronal differentiation (Yang et al. 1998; Ben Zablah et al. 2021; Villalonga et al. 2023). Consistently, the mean F-actin intensity decreased significantly after knockdown of either the *EIciLIMK1* or *LIMK1* mRNA (Fig. 7J; Supplemental Fig. S13I,J). Taken together, these data suggested that SRSF1 could suppress the biogenesis of *EIciLIMK1* and that the expression level of SRSF1 decreased to release this suppression during neuronal differentiation. *EIciLIMK1* regulated the expression of its parental gene in *cis*, and up-regulated LIMK1 led to increased levels of p-cofilin, which facilitated the formation of F-actin during neuronal differentiation.

## Discussion

**Figure 8.**
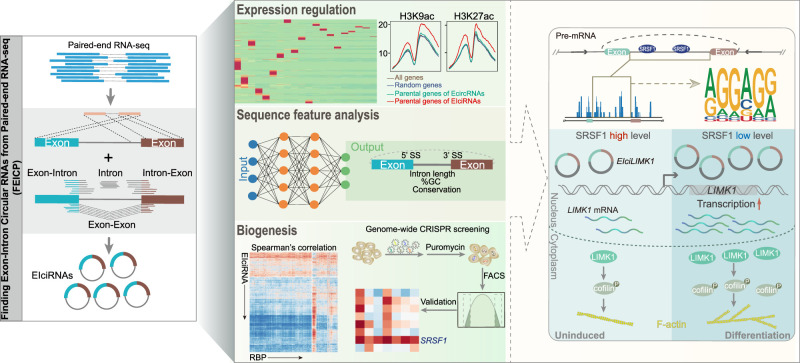
An illustrated summary of this study including the FEICP pipeline, features of EIciRNAs and CIRs, EIciRNA biogenesis, and functional relevance of EIciRNAs.

We developed FEICP, a computational framework that efficiently detects CIR from short-read paired-end RNA-seq data. Although HTS and bioinformatic improvements have made it possible to characterize circRNAs in detail from short-read and long-read RNA-seq, the general features and biological implications of EIciRNAs have been less investigated largely owing to the absence of a reliable computational pipeline for detection. FEICP enables the systematic identification of EIciRNAs in tissues and cells (Fig. 8). EIciRNAs possess sequence features in flanking introns, composed exons, and CIRs (Fig. 8). Features in the expression patterns and functional roles of EIciRNAs were also analyzed (Fig. 8). Based on bioinformatic analyses and CRISPR screening with reporters, SRSF1 has been identified to inhibit the biogenesis of a portion of EIciRNAs by binding to CIRs (Fig. 8). During neuronal differentiation, SRSF1 is down-regulated, and correspondingly, *EIciLIMK1* is up-regulated; moreover, *EIciLIMK1* plays a *cis* role in promoting the expression of *LIMK1* to enhance neuronal differentiation (Fig. 8).

ONT is a powerful tool for sequencing the full length of circRNAs, although it still has drawbacks such as a lower sequencing depth than HTS (Byrne et al. 2019; Liu et al. 2021; Rahimi et al. 2021; Xin et al. 2021; Zhang et al. 2021). In addition, ONT methods for circRNAs generally rely on rolling circle cDNA amplification; thus, shorter circRNAs are more prone to be captured than longer circRNAs. This was confirmed through comparing the overall length of EIciRNAs detected by FEICP and isoCirc (Supplemental Fig. S1D). HTS is still the major tool for identifying circRNAs. The FEICP workflow combines the detection of BSJs and CIRs with stringent criteria to remove potential false-positive results for EIciRNAs. Comparisons between FEICP and the other tools, including isoCirc, CIRI-full, and CYCLeR, showed that FEICP was a valuable tool in the comprehensive characterization of EIciRNAs based on HTS data. A substantial number of EIciRNAs were uniquely detected by each method, showing distinct features of each method.

Previously, little was known about the sequence features of EIciRNAs as a subclass of circRNAs, except that their sequences are composed of both exonic and intronic sequences. In this study, we revealed that the flanking introns, CIRs, and exonic sequences of EIciRNAs all have characteristics distinct from those of EcircRNAs. CIRs show several key distinctions from LIRs, which have been subjected to in-depth investigations for a long period of time (Middleton et al. 2017; Schmitz et al. 2017; Wang et al. 2020; Yeom et al. 2021). Therefore, CIRs and LIRs can be defined as two types of IR based not only on whether they are retained in circular or linear transcripts.

In addition to sequence properties, functional insights have been provided in this study. EIciRNAs localize primarily in the nucleus, and parental genes of EIciRNAs tend to be actively transcribed. Histone modifications and DNase-sensitive sites that are linked to gene activation are significantly enriched in genes that give rise to EIciRNAs. Therefore, on a genome-wide scale, EIciRNAs are associated with active gene expression. CIRs tend to localize at the 5′-end of the transcript, and U1 snRNP was previously found to be recruited by the 5′ SS of CIR to promote transcription in *cis* (Li et al. 2015). The expression of EIciRNAs is highly tissue specific, and EIciRNA-generating is associated with marks of active gene expression; therefore, EIciRNAs may be key elements in a positive feedback mechanism that ensures specific and active expression of parental genes. However, the positive association between EIciRNA generation and active transcription of its parental gene and the causal relationship needs further investigation. However, for some particular genes, correlation in levels of EIciRNAs and mRNAs may be determined by complex or upstream regulatory mechanisms and not just dictated by the transcriptional feedback of EIciRNAs. One EIciRNA, *circRNF217,* was reported to play a *trans* role as a sponge of *miR-130-3p* in the cytoplasm to promote antibacterial responses in teleost fish (Zheng et al. 2022). Our data showed that the majority of EIciRNAs localize to the nucleus and tend to play a *cis* role, although this does not exclude the possibility that some EIciRNAs can play *trans* roles. On the other hand, LIRs in mRNAs generally contain premature termination codons (PTCs), and these mRNAs can be recognized by nonsense-mediated decay (NMD), which results in mRNA degradation (Monteuuis et al. 2019). LIRs tend to localize to the 3′-end of transcripts and are associated with negative regulation, whereas CIRs tend to localize to the 5′-end of transcripts and are potentially associated with positive regulation of gene expression. The distribution of CIRs in the 5′ region could also be explained by the general tendency of 5′ localization of circRNAs (Guo et al. 2014; Westholm et al. 2014; Rybak-Wolf et al. 2015), and the *cis*-acting role of EIciRNAs needs further evidence.

The biogenesis of EIciRNAs should be subjected to strict and complex regulation to ensure high tissue specificity and respond to cellular dynamics such as differentiation. An array of RBPs is involved in regulating EIciRNA expression, and most EIciRNAs are negatively regulated by RBPs; vice versa, the majority of these RBPs play suppressive roles in the expression of EIciRNAs. SRSF1 was identified by whole-genome screening as an inhibitor of EIciRNA biogenesis, at least for a significant portion of EIciRNAs, by binding to CIRs. Metaprofiles of SRSF1 binding to the EIciRNAs showed that SRSF1 bound to both the retained introns and circularizing exons, whereas no obvious difference in SRSF1 binding was observed among the three groups of EIciRNAs (Supplemental Fig. S11H). However, further investigations are needed to understand the complex effects of SRSF1, and one particular point would be to examine its cofactors and their subsequent roles in promoting or suppressing the generation of EIciRNAs. The reporters used in the screening were aimed at only identifying factors that bind to intronic sequences of CIRs, and the complete picture of EIciRNA biogenesis requires further studies. Consistent with the findings of previous studies (Middleton et al. 2017; Monteuuis et al. 2019; Chen et al. 2022b), we also showed that SRSF1 was involved in regulating LIRs, and there was limited overlap between regulated CIR and LIR events for both introns and parental genes (Fig. 6D). These findings further indicated the role of SRSF1 in regulating IR and indicated that the regulation of CIRs and LIRs might be distinct. In neuronal differentiation, SRSF1 also plays a negative role by regulating EIciRNA biogenesis, although SRSF1 can use combined functional mechanisms. In the example of *EIciLIMK1*, its expression is suppressed by SRSF1, and during neuronal differentiation, decreased SRSF1 levels result in up-regulated *EIciLIMK1* expression. The activation pathway of *EIciLIMK1–LIMK1–*p-cofilin*–*F-actin is recognized to promote neuronal differentiation.

## Methods

An extended version of the Methods with full descriptions of cell culture, experimental details, data processing, and bioinformatic analyses is provided as a Supplemental Methods section.

### The FEICP pipeline

To identify EIciRNAs from paired-end HTS data, the FEICP pipeline was developed, which was composed of the following three steps: (1) circRNA annotation, (2) detection of exon–intron junctions or intron sequences from paired reads of the annotated BSJ, and (3) annotation of EIciRNAs and retained introns. These steps were illustrated as following in detail. In step 1, paired-end RNA-seq reads were mapped onto the reference genome using BWA-MEM 0.7.17-r1198-dirty (Li 2013) with the parameter -T 19, as suggested by CIRI2 pipeline (Gao et al. 2018). CircRNAs were detected from the alignments using CIRI2.pl, with the GTF file from GENCODE as gene annotation. In step 2, circRNAs with “circRNA_type” being “exon” in the results of CIRI2 were selected, and their coordinates were intersected with the transcripts from gene annotation using BEDTools (Quinlan and Hall 2010). Each circRNA was scored based on its annotated transcripts as follows: (1) score was two if both start and end coordinates of the circRNA matched to the exon boundary of a transcript; (2) score was one if only one start or end coordinate of the circRNA matched to the exon boundary of a transcript; and (3) score was zero if neither start nor end coordinate of the circRNA matched to any exon boundary of a transcript. Transcripts with the maximal score were selected as the host transcripts of the circRNA. Then all reads containing BSJs were extracted using seqtk v1.3-r116-dirty (https://github.com/lh3/seqtk) and were mapped onto the reference genome sequences of circRNAs. CircRNAs with BSJ reads exclusively mapping within introns or across exon–intron junctions with at least a 5-nt overhang were considered as potential EIciRNAs and used for further analysis. In step 3, IR was validated using the widely used method for the detection of IRs in linear transcripts (Braunschweig et al. 2014; Middleton et al. 2017; Yeom et al. 2021). The raw sequencing reads were firstly mapped onto the reference genome using the splice-aware aligner STAR v 2.7.5a (Dobin et al. 2013). Based on the alignments, several metrics for each intron were computed, including the counts of reads mapping to exon–exon (#EE), exon–intron (#EI), and intron–exon (#IE) junctions; the middle 200 nt of the intron (or the full intron, if <200 nt; #I); and read coverage of the intron (#IC). All the above junctions must have an overhang no less than 5 nt. An intron was considered to be retained in circRNA from step 2 only when it fulfilled the following requirements: #EE ≥ 1, #EI ≥ 1, #IE ≥ 1, #I ≥ 1, and #IC ≥ 0.9. And, last, circRNAs detected in step 2 were filtered based on the conditions in step 3 to obtain EIciRNAs. The counts of BSJs that met the conditions in step 2 were used as EIciRNA counts.

### Public data sets

The public data sets used in this study can be found in Supplemental Table S10.

## Data access

The list of all oligonucleotide sequences used in this study are available as Supplemental Table S11. All raw sequencing data generated in this study have been submitted to the NCBI Gene Expression Omnibus (GEO; https://www.ncbi.nlm.nih.gov/geo/) under accession numbers GSE241685 and GSE253233. The FEICP computational pipeline has been deposited in GitHub (https://github.com/xjyx/FEICP) and also uploaded as Supplemental Code along with other custom scripts.

## Supplementary Material

Supplement 1

Supplement 2

Supplement 3

Supplement 4

Supplement 5

Supplement 6

Supplement 7

Supplement 8

Supplement 9

Supplement 10

Supplement 11

Supplement 12

Supplement 13
