# Supplementary material for: Systematic identification and characterization of exon–intron circRNAs
Source: Genome Res. 2024 Mar;34(3):376–93. doi: 10.1101/gr.278590.123 (PMC11067877; doi:10.1101/gr.278590.123)
Supplement: Supplement 13 [file Supplemental_Materials.pdf]

Supplementary Materials for

Systematic identification and characterization of exon-intron circRNAs

Yinchun Zhong[1, #], Yan Yang[2, #], Xiaolin Wang[2], Bingbing Ren[3], Xueren Wang[4, 5, *], Ge Shan[2, *], Liang Chen[1, *]

1. Department of Cardiology, The First Affiliated Hospital of USTC, Division of Life Science and Medicine, University of Science and Technology of China, Hefei, 230027, China

2. Hefei National Laboratory for Physical Sciences at Microscale, Department of Clinical Laboratory, The First Affiliated Hospital of USTC, School of Basic Medical Sciences, Division of Life Science and Medicine, University of Science and Technology of China, Hefei, 230027, China

3. Department of Pulmonary and Critical Care Medicine, Regional Medical Center for National Institute of Respiratory Diseases, Sir Run Run Shaw Hospital, School of Medicine, Zhejiang University, Hangzhou, 310016, China

4. Department of Anesthesiology, Shanxi Bethune Hospital, Taiyuan, 030032, China

5. Department of Anesthesiology, Tongji Hospital, Tongji Medical College, Huazhong University of Science and Technology, Wuhan, 430030, China.

This PDF file includes:

Supplemental Figures S1-S13

Descriptions of Supplemental Tables S1-S11

Supplemental Methods

References for Supplemental Materials

Supplemental Figure S1

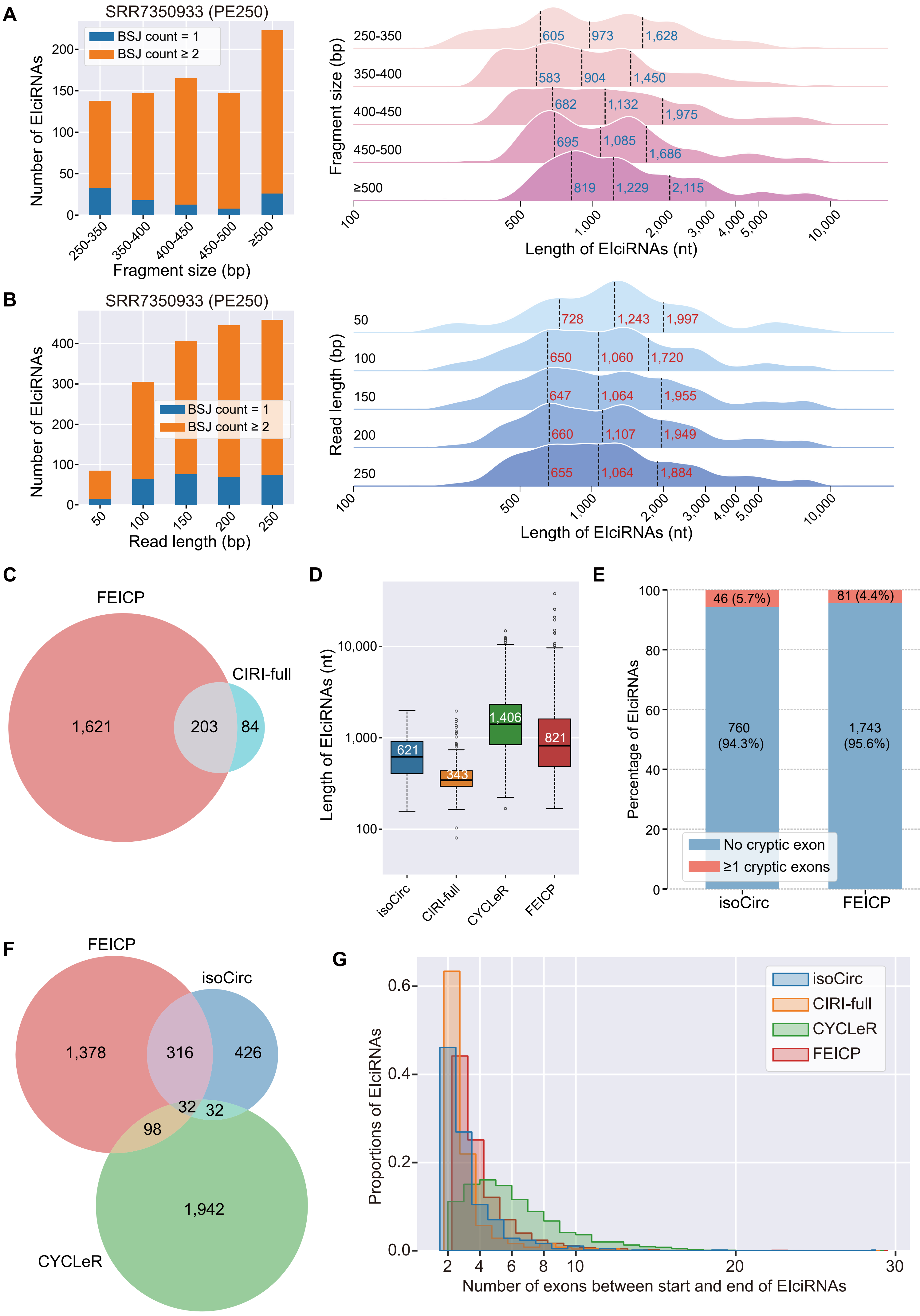

Supplemental Figure S1. Performance of FEICP.

A. EIciRNA number (left) and length distribution (right) identified on sequencing reads with different fragment size. The dashed lines in the right represent the 25%, 50%, and 75% quantiles of EIciRNA lengths. **B**. EIciRNA number (left) and length distribution (right) identified on sequencing reads with different length trimmed from PE250 data. The dashed lines in the right represent the 25%, 50%, and 75% quantiles of EIciRNA lengths. **C**. Venn diagram showing the overlap of identified EIciRNAs between FEICP and CIRI-full in HEK293 cells. **D**. Boxplots showing the distribution of EIciRNA length identified by isoCirc, CIRI-full, CYCLeR, and FEICP in HEK293 cells, with the median length labeled for EIciRNAs in each group. **E**. Stacked bar plots showing the proportion of intron-retaining circRNAs, detected using FEICP or isoCirc, that generate isoforms with or without cryptic exons. **F**. Venn diagram showing the overlap of EIciRNAs identified by FEICP, isoCirc, and CYCLeR in HEK293 cells. **G**. Histogram showing the distribution of exon numbers between the start and end exons of EIciRNAs identified by different pipelines.

Supplemental Figure S2

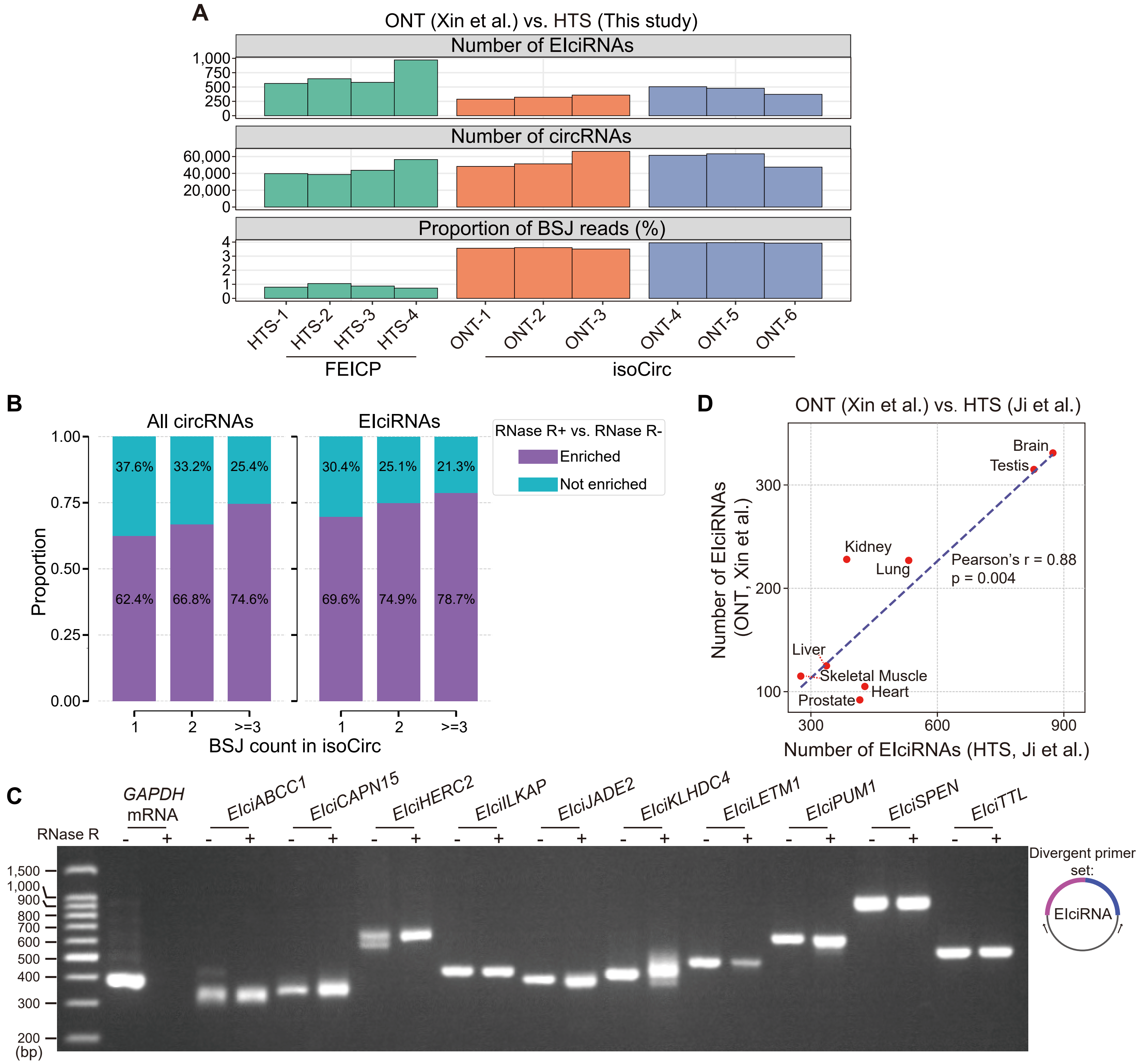

Amplicon (bp)	325	341	605	421	385	417	464	615	986	542
EIciRNA length (nt)	3,495	2,197	2,393	1,842	2,226	3,880	2,239	2,831	3,863	1,976

Supplemental Figure S2. Comparison of FEICP with isoCirc.

A. Number of EIciRNAs, circRNAs, and proportions of back-splicing junction (BSJ) reads detected from HTS data in our study and published ONT datasets in HEK293 cells. HTS, high-throughput sequencing; ONT, Oxford Nanopore Technology sequencing. **B**. Stacked bar plots showing the proportion of isoCirc-detected circRNAs and EIciRNAs with or without enrichment in HTS data by RNase R treatment in HEK293 cells. Public total RNA-seq data downloaded from NCBI (SRA: SRR22315104, SRR22315105, SRR22315106, and SRR22315107) were generated with RNase R- samples. EIciRNAs with foldchange ≥ 1.5 and P-value < 0.05 were considered as enriched by RNase R. P-values were calculated using the Student's *t*-test. **C**. RT-PCR validated 10 EIciRNAs identified by FEICP but missed out by isoCirc in HEK293 cells (Supplemental Table S2). RNase R was used to digest linear RNAs, and *GAPDH* mRNA was used as a control for RNase R treatment. Both the lengths of RT-PCR amplicon and EIciRNAs were indicated for each EIciRNA. Divergent primers with both ends located in the retained introns were used to amplify EIciRNAs. **D**. Correlation of EIciRNA number from published HTS or ONT datasets of eight human tissues.

Supplemental Figure S3

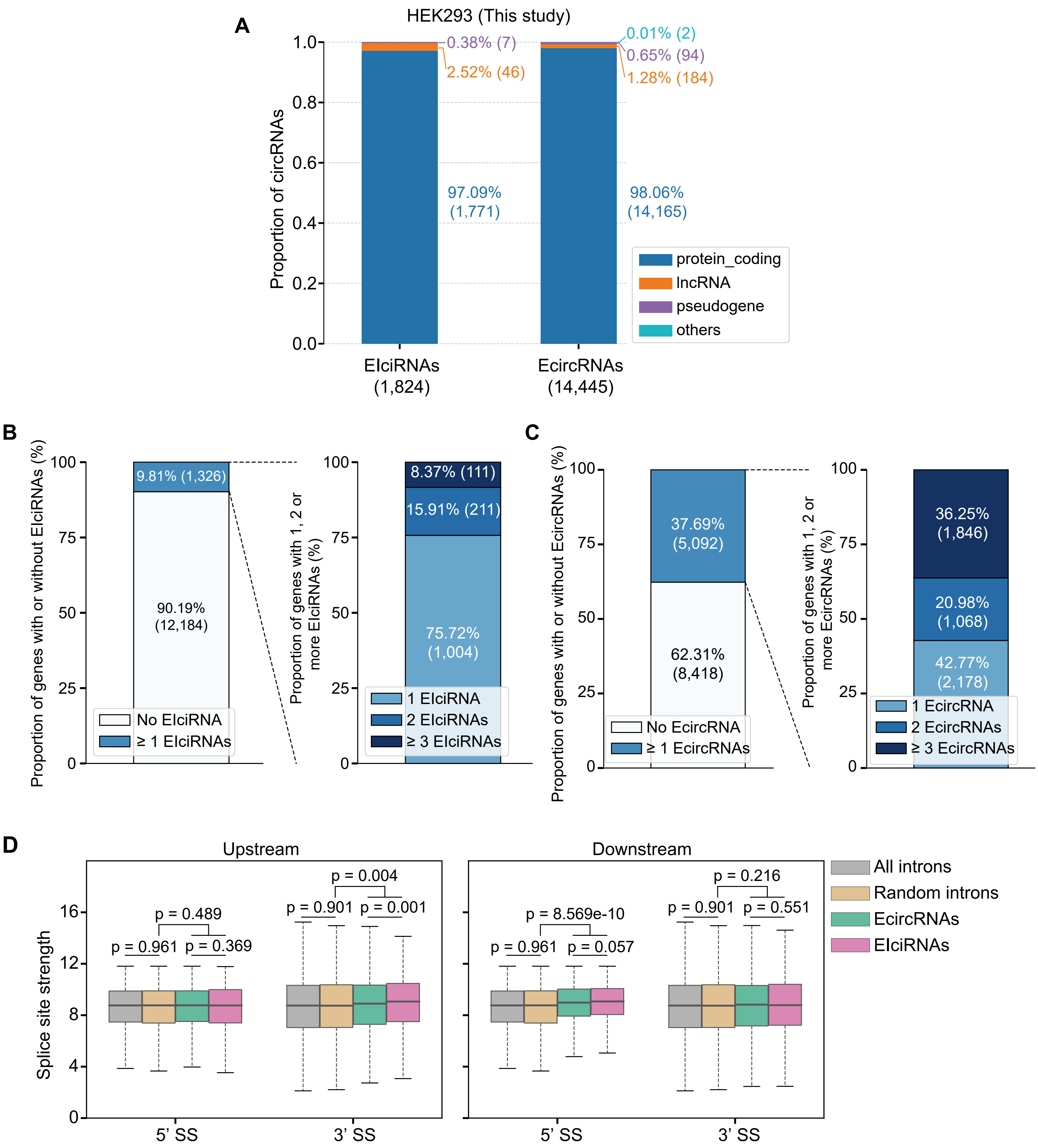

Supplemental Figure S3. Features of EIciRNAs.

A. Distribution of parental gene types of EIciRNAs and EcircRNAs detected in HEK293 cells. **B**. Percentage of EIciRNA parent genes (left) and genes that give rise to 1, 2 or more EIciRNAs (right). **C**. Percentage of EcircRNA parent genes (left) and genes that give rise to 1, 2 or more EcircRNAs (right). **D**. Boxplots showing the 5′ and 3′ splice-site strength of flanking introns of EcircRNAs and EIciRNAs detected in HEK293 cells. All introns annotated in the human genome and randomly selected human introns were used as controls. P-values were calculated using the Wilcoxon rank-sum test.

Supplemental Figure S4

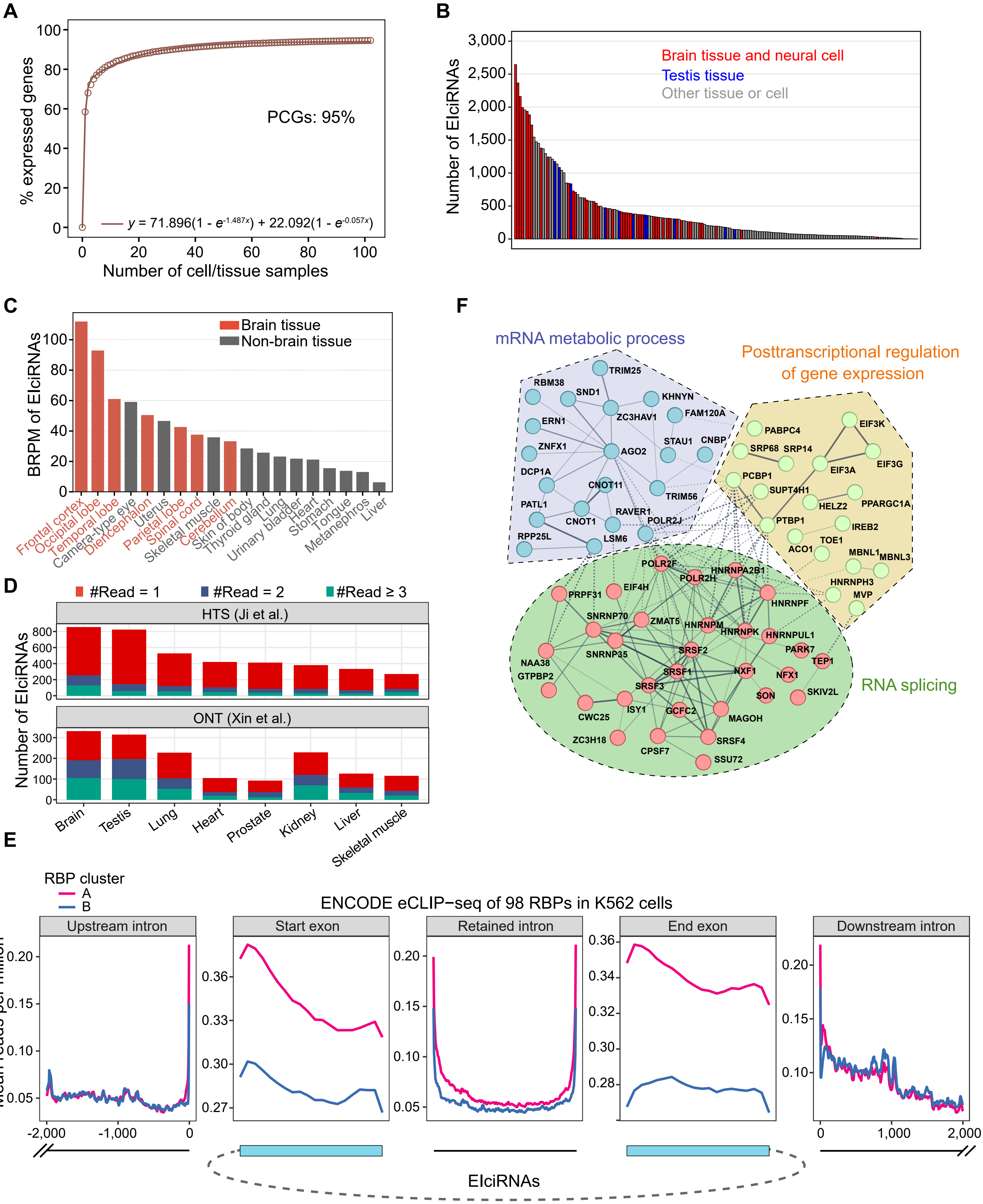

Supplemental Figure S4. Expression features of EIciRNAs across human tissues.

A. The percentage of expressed human protein-coding genes (PCGs) with the accumulating number of cell or tissue samples. The circles represented the mean values from 1,000 iterations and exponential curve fitting was applied. The corresponding equation and limit value were shown. **B**. The number of EIciRNAs detected from 244 published HTS datasets in 102 human cell or tissue samples. Brain tissues or neural cells were indicated as red, and testis tissues were indicated as blue. **C**. Bar plots displaying EIciRNA levels from ENCODE HTS data of 19 human fetal tissues. 12 non-brain tissues and 7 brain tissues were labeled as grey and red, respectively. BRPM, backspliced reads per million. **D**. The number of EIciRNAs identified in published HTS or ONT datasets of eight human tissues. **E**. Metaplots displaying the mean binding density of 80 RBPs in “Cluster A” and 18 RBPs in “Cluster B” on the retained introns, circular exons, and flanking introns of 1,231 “Cluster 1” EIciRNAs. These clusters were depicted in Fig. 2E. **F**. STRING analysis of protein-protein interaction network for top 100 RNA-binding proteins (RBPs) with the most negative correlations to EIciRNAs in 19 human fetal tissues. The network was grouped into 3 clusters using k-means clustering algorithm, and the top-ranked enriched GO term of RBP genes was shown for each group.

Supplemental Figure S5

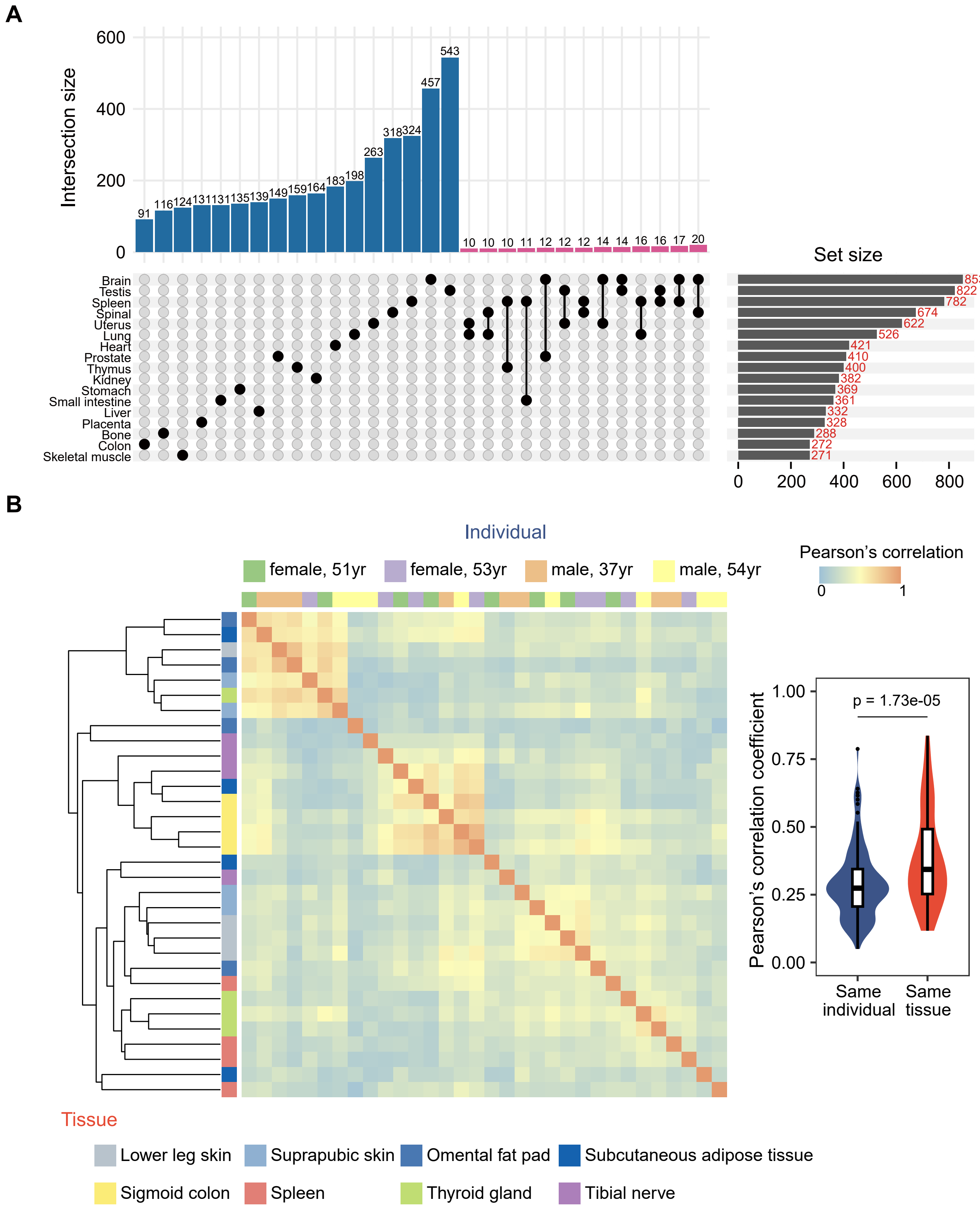

Supplemental Figure S5. Tissue specificity of EIciRNAs across human tissues.

A. UpSet plot showing the overlap of EIciRNAs identified in published HTS datasets of 17 human tissues. Intersections with no less than 10 EIciRNAs were shown. **B**. Correlation matrix of EIciRNA levels across samples in 8 tissues from four individual human beings. Row annotations indicate different tissues, and column annotations indicate different individuals (left). Violin plots showed the Pearson's correlation coefficients of EIciRNA levels among the same tissue type from distinct individuals or the same individual in different tissues (right). P-value was calculated using the Wilcoxon rank-sum test.

Supplemental Figure S6

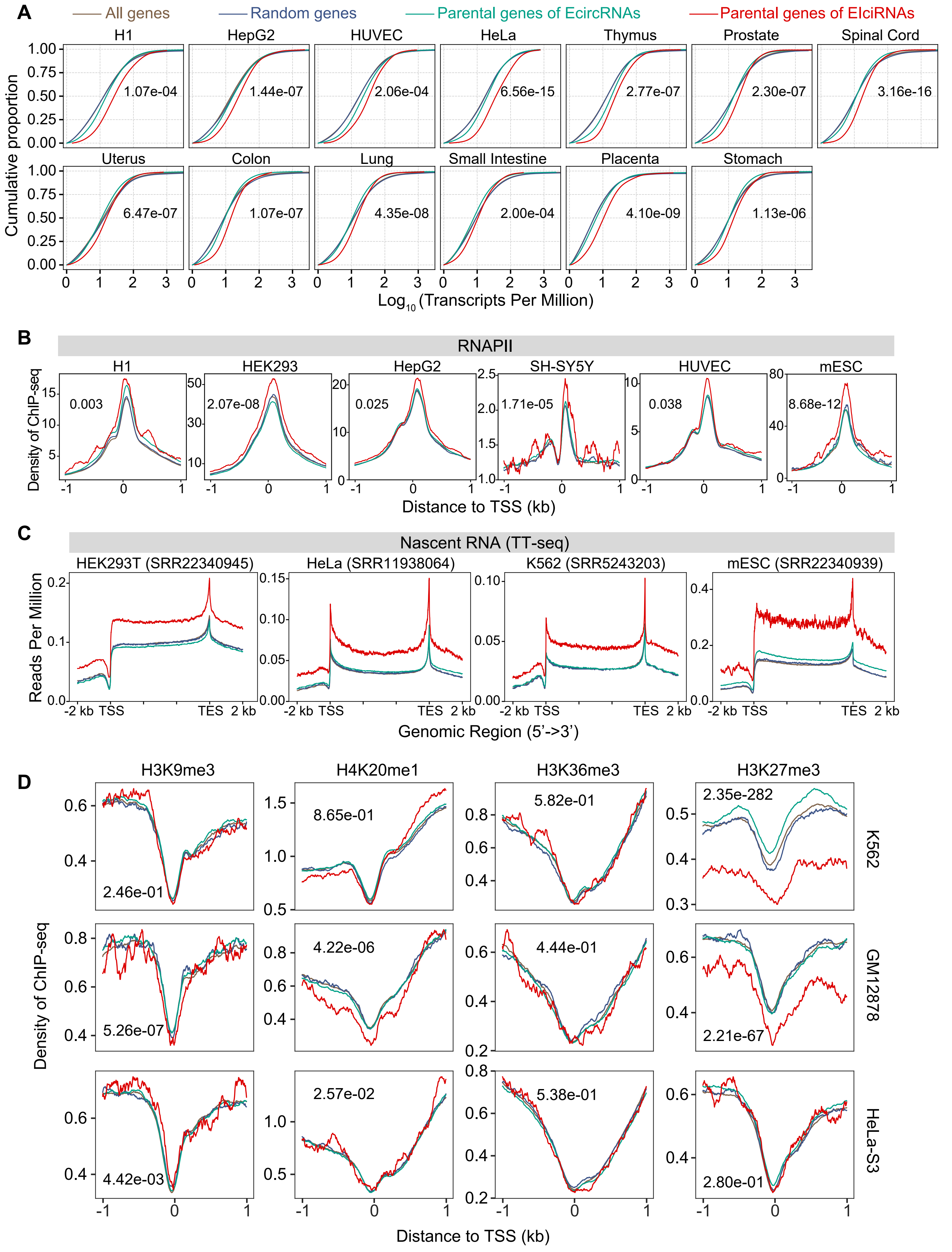

Supplemental Figure S6. Expression and epigenetic regulation of EIciRNA parent genes. **A**. Cumulative distribution of expression levels (TPM) of all genes, random genes, parental genes of EcircRNAs, and parental genes of EIciRNAs in 4 human cell lines and 9 human tissues. HUVEC, human umbilical vein endothelial cells; All genes, all expressed protein-coding genes (PCGs) with TPM $\geq$ 1; Random genes, 2,000 genes randomly selected from all genes; Parental genes of EIciRNAs, PCGs generating EIciRNAs; Parental genes of EcircRNAs, PCGs generating EcircRNAs but no EIciRNAs; TPM, transcripts per million. **B**. Distributions of RNAPII ChIP-seq signals around the TSS regions of the indicated groups in 6 cell lines. TSS, transcription start site. **C**. Metagene plots showing the coverage (RPM) of TT-seq along the whole gene body regions of the indicated groups in 4 cell lines. RPM, reads per million. **D**. ChIP-seq signals of H3K9me3, H4K20me1, H3K36me3, and H3K27me3 around the regions of the indicated groups of genes in K562, GM12878, or HeLa-S3 cell lines. In A, B, and D, P-values were calculated with the Wilcoxon rank-sum test for the comparison between parental genes of EIciRNAs and parental genes of EcircRNAs.

Supplemental Figure S7

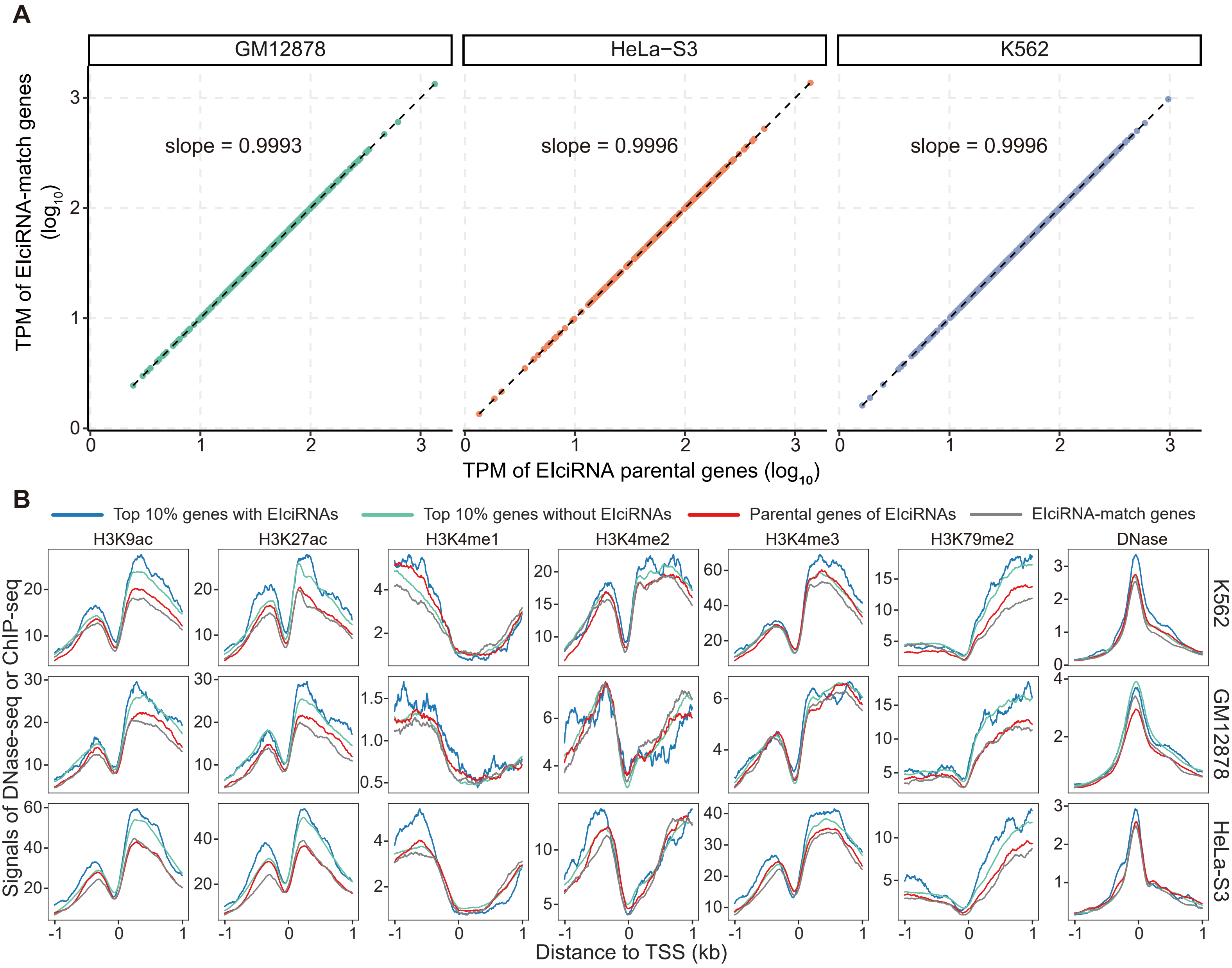

Supplemental Figure S7. Features of Expression and epigenetic marks of genes with or without EIciRNA generation.

A. Scatter plots displaying the correlation between the expression levels of EIciRNA parental genes and those of "EIciRNA-match" genes that do not generate EIciRNAs in GM12878, HeLa-S3, and K562 cells. The slope of the fitted line of linear regression was indicated for each cell line. For each EIciRNA, the gene with the closest expression level to its parental gene was referred to as its "EIciRNA-match" gene. **B**. ChIP-seq signals of H3K9ac, H4K27ac, H3K4me1, H3K4me2, H3K4me3, H3K79me2 and DNase around the TSS regions of the indicated groups of genes in K562, GM12878, or HeLa-S3 cell lines. The top 10% of genes with the highest expression levels were divided into two groups: genes generating EIciRNAs ("Top 10% genes with EIciRNAs"), and genes generating no EIciRNAs ("Top 10% genes without EIciRNAs").

Supplemental Figure S8

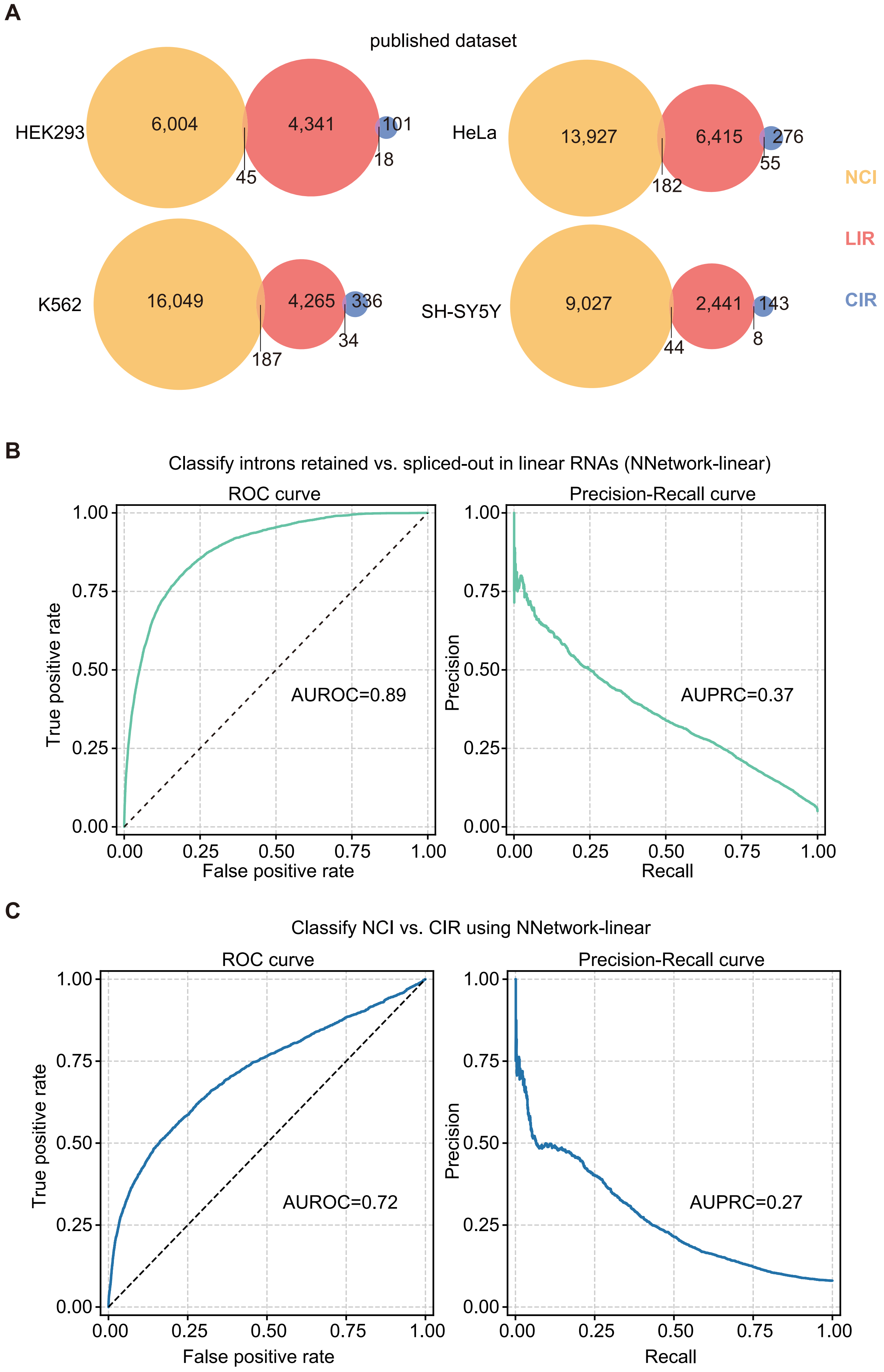

Supplemental Figure S8. Classification and prediction of introns.

A. Venn diagrams showing the overlap of NCI, LIR, and CIR detected from the published HTS datasets of HEK293, HeLa, K562, and SH-SY5Y cell lines. LIR was detected from poly(A)-plus RNA-seq data through IRFinder with the cutoff (IRratio ≥ 0.1). CIR was detected from RNase R-treated RNA-seq data using FEICP with the cutoff (PIR ≥ 0.1). NCI represented spliced introns of EcircRNAs with the cutoff (PIR ≤ 0.02). PIR, percent intron retention. **B**. The ROC curve (left) and precision-recall curve (right) illustrating the performance of the neural network predicting intron retention in linear RNAs (NNetwork-linear). The AUROC (area under the ROC curve) and AUPRC (area under the precision-recall curve) values are shown. **C**. The ROC curve (left) and precision-recall curve (right) illustrating the performance of the NNetwork-linear in predicting intron retention in circular RNAs.

Supplemental Figure S9

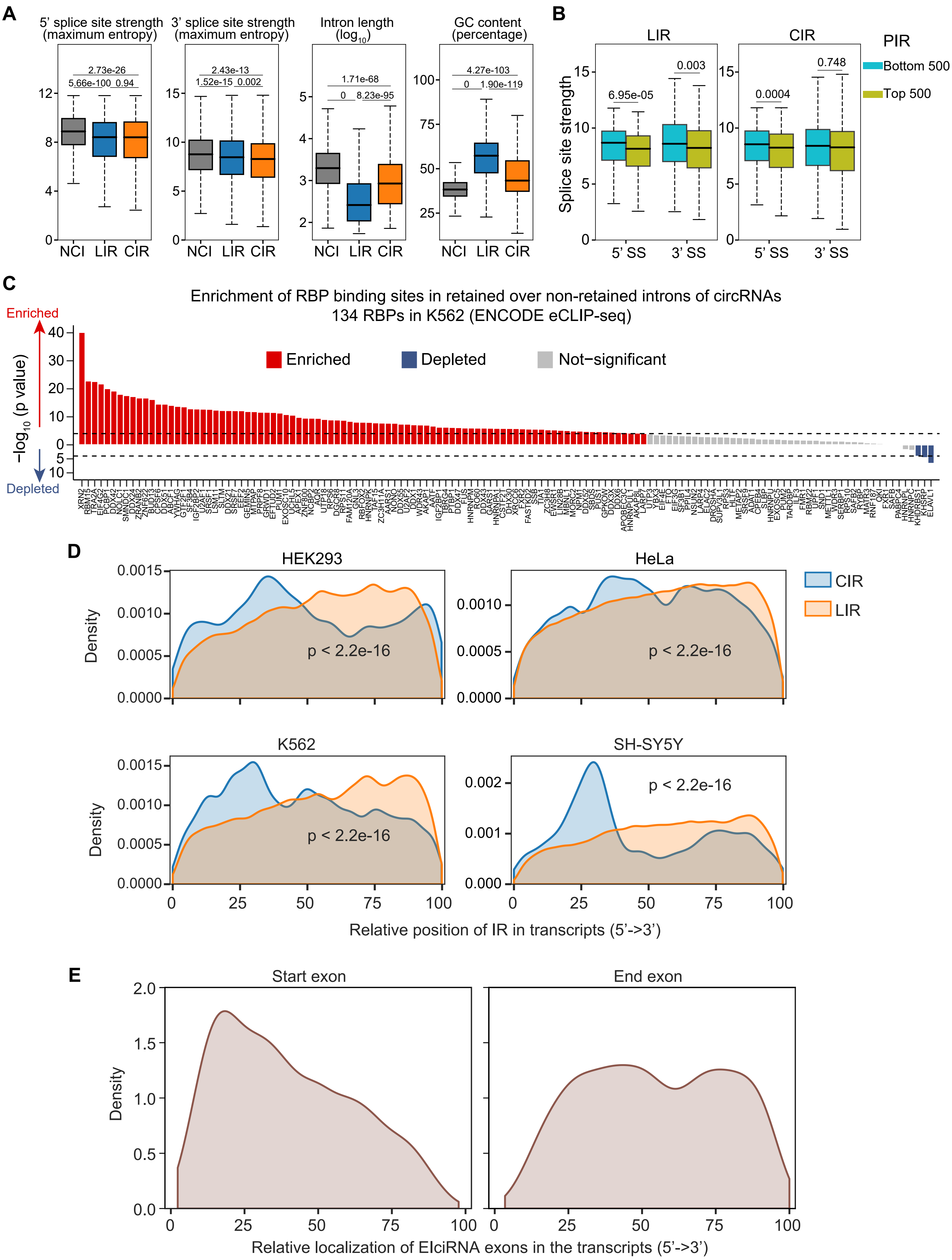

Supplemental Figure S9. Features of CIR.

A. Boxplots displaying 5′ splice-site strength, 3′ splice-site strength, intron length and GC content of NCI, LIR and CIR detected in our HEK293 dataset. **B**. Boxplots displaying the 5′ and 3′ splice-site strength of 500 LIR (CIR) with the highest PIR and 500 LIR (CIR) with the lowest PIR. PIR, percent intron retention. **C**. RBP binding enrichment in CIR versus NCI from 134 RBP eCLIP-seq datasets in ENCODE. **D**. Density curves showing the relative localization of LIR and CIR along their host transcripts from the published HTS datasets of HEK293, HeLa, K562 and SH-SY5Y cells. **E**. Density curves showing the relative localization of start and end exons of EIciRNAs along their host transcripts in HEK293 cells. For A-D, P-values were calculated using the Wilcoxon rank-sum test.

Supplemental Figure S10

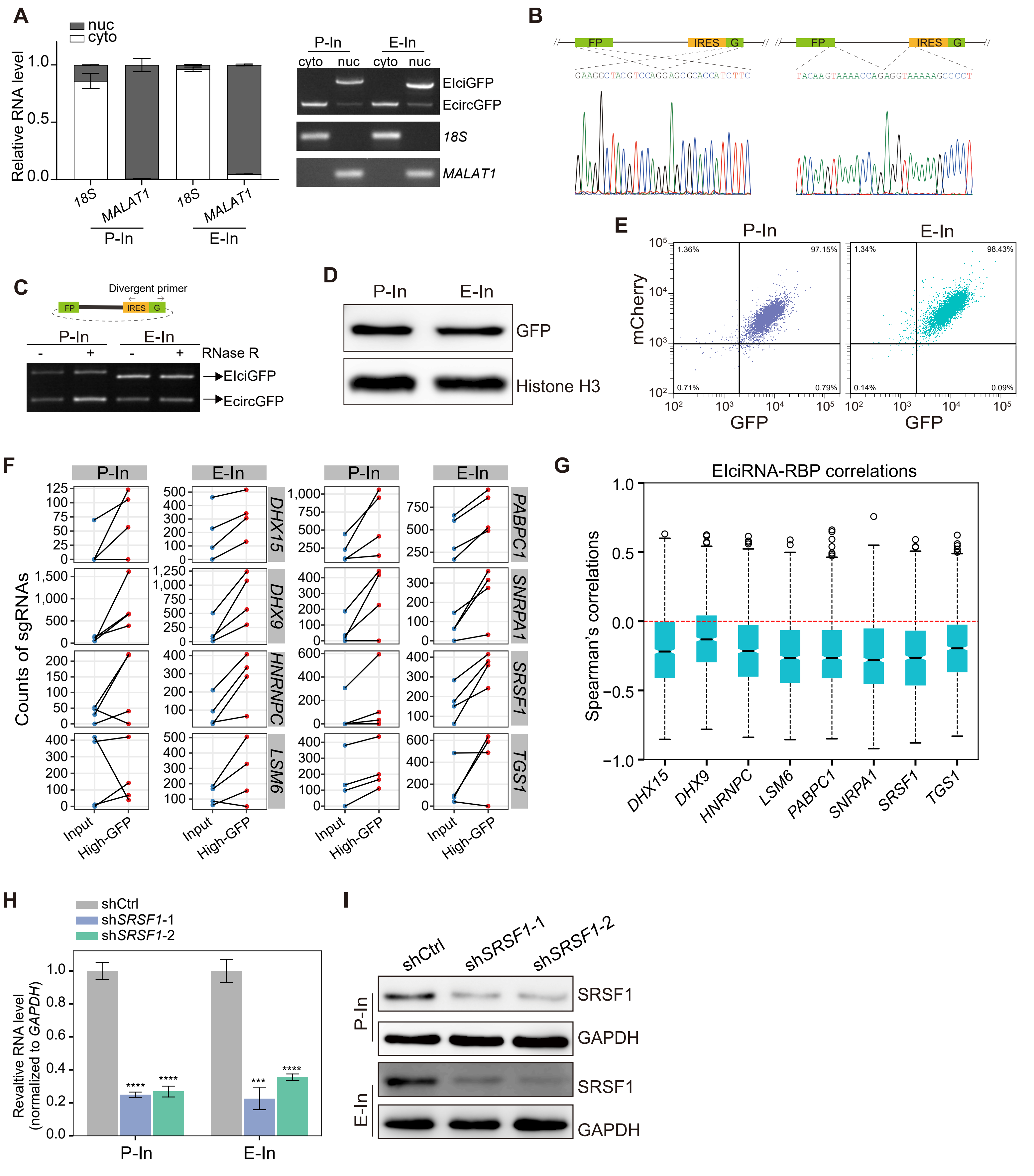

Supplemental Figure S10. Genome-wide CRISPR screening identifies SRSF1 as a regulator of EIciRNA biogenesis.

A. Stacked bar plots showing the distribution of nuclear and cytosolic markers assessed by RT-qPCR in the cytosol and nucleus obtained through nucleocytoplasmic separation in P-In and E-In cells (left). RT-PCR analysis of EIciGFP and EcircGFP distribution in the nuclear and cytoplasmic fraction of P-In and E-In cells (right). **B**. Sanger sequencing of BSJ sequences (left) and linear splicing junction sequences (right) for RT-PCR products of circGFP in P-In and E-In cells. **C**. RT-PCR showing the expression of EIciGFP and EcircGFP in P-In and E-In cells. RNase R was used for the digestion of linear RNAs. Divergent primers were used to amplify EIciGFP or EcircGFP. **D**. Western blot showing the expression of GFP protein in P-In and E-In cells, respectively. Histone H3 was used as the loading control. **E**. Flow cytometric analysis showed that ~97% of cells co-expressed GFP and mCherry in P-In and E-In cells. **F**. Counts of four sgRNAs for each of eight genes between input and high-GFP groups in P-In and E-In cells. **G**. Boxplots showing the Spearman's correlations, defined in Fig. 2E, between EIciRNAs and 8 RBPs. **H**-**I**. RT-qPCR (**H**) and western blots (**I**) showing the knockdown efficiency of *SRSF1* in P-In and E-In cells. shCtrl, shRNA with scrambled sequences; sh*SRSF1*-1 and sh*SRSF1*-2, two independent shRNAs against *SRSF1*. For A and H, error bars represent standard deviation (SD) in triplicate experiments, and P-values were calculated using two-tailed Student's *t*-test. ***$p < 0.001$; ****$p < 0.0001$.

Supplemental Figure S11

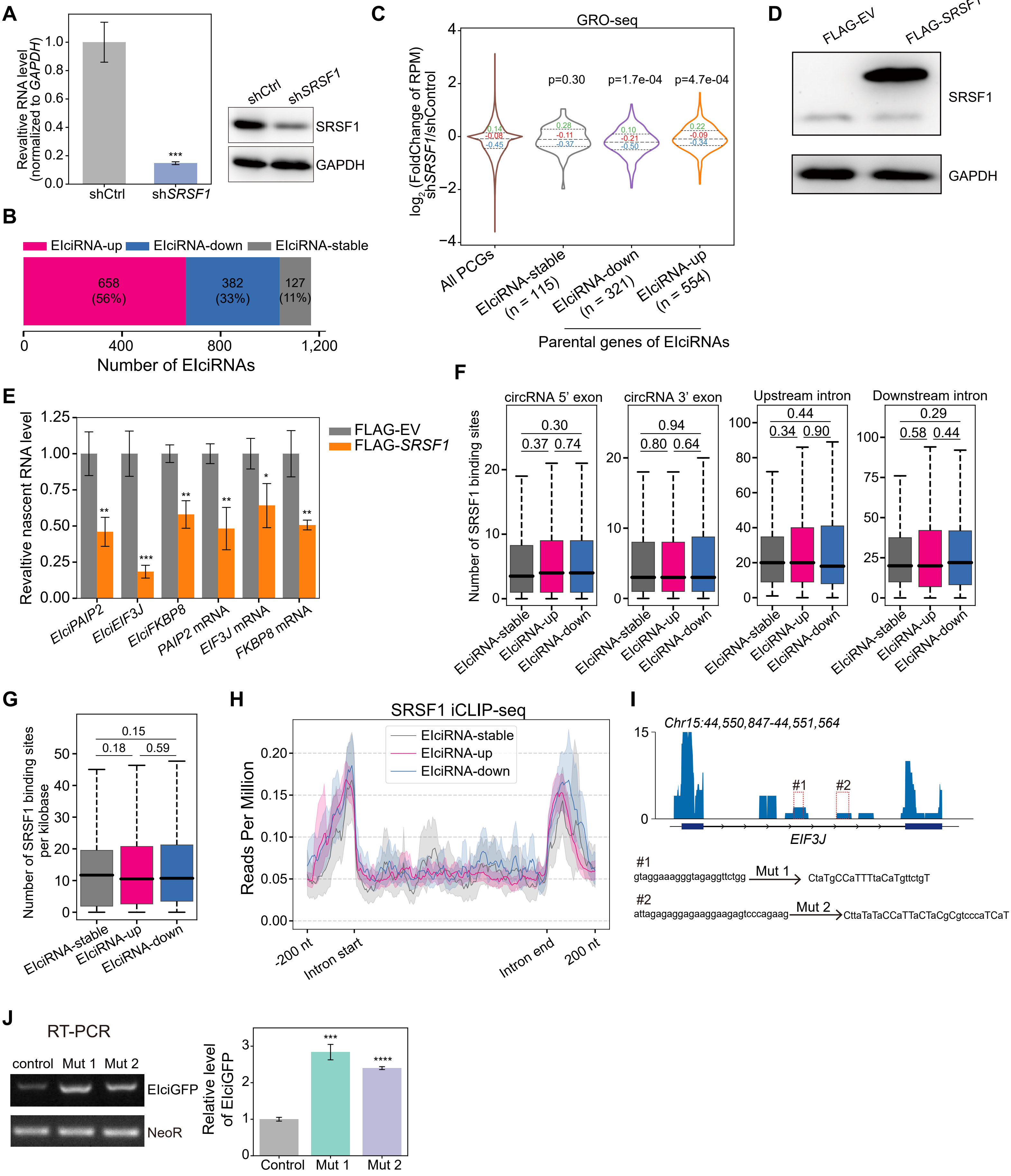

Supplemental Figure S11. SRSF1 suppresses the biogenesis of a portion of EIciRNAs.

A. RT-qPCR and western blots showing *SRSF1* knockdown in HEK293 cells. shCtrl, shRNA with scrambled sequences; sh*SRSF1*, shRNA against *SRSF1*. **B**. Stacked bar plot showing the proportion of the indicated EIciRNAs. EIciRNA-up, up-regulated EIciRNAs upon *SRSF1* knockdown in HEK293 cells; EIciRNA-down, down-regulated EIciRNAs upon *SRSF1* knockdown in HEK293 cells; EIciRNA-stable, unaltered EIciRNAs upon *SRSF1* knockdown in HEK293 cells. **C**. Violin plots displaying the nascent RNA foldchanges of genes in indicated groups upon *SRSF1* knockdown in HEK293 cells. P-values between all protein-coding genes and genes in the EIciRNA-stable, EIciRNA-down, EIciRNA-up group were calculated and indicated. The 25th, 50th, and 75th percentile of the foldchanges was labelled in blue, red, and green, respectively. **D**. Western blot showing the SRSF1 protein level when *SRSF1* was overexpressed in HEK293 cells. GAPDH was used as the loading control. FLAG-EV, empty vector. **E**. RT-qPCR analysis of nascent levels for three EIciRNA / mRNA pairs after *SRSF1* overexpression in HEK293 cells. **F**. Boxplots displaying the counts of SRSF1 binding sites in flanking introns and internal exons of the indicated groups of EIciRNAs. **G**. Boxplots showing the density of SRSF1 binding sites in CIR of the indicated groups. **H**. Metaplots displaying the binding density of SRSF1 on the indicated groups of EIciRNAs. **I**. IGV snapshot showing the SRSF1 iCLIP-seq signals in the *EIciEIF3J* locus. Two SRSF1 binding GA-rich regions in the retained intron of *EIciEIF3J* were framed as red dotted lines, and labeled as #1 and #2, respectively. **J**. Semi-quantitative RT-PCR gels and the quantification of EIciGFP levels in HEK293 cells after transfection of indicated mutation plasmids. The eukaryotic resistance gene (NeoR) of the plasmids was used as the loading control. For A, E-G, and J, P-values were calculated using two-tailed Student's *t*-test. *$p < 0.05$; **$p < 0.01$; ***$p < 0.001$; ****$p < 0.0001$. In C, P-values were calculated using the Kolmogorov-Smirnov test. In A, E, and J, error bars represent SD in triplicate experiments.

Supplemental Figure S12

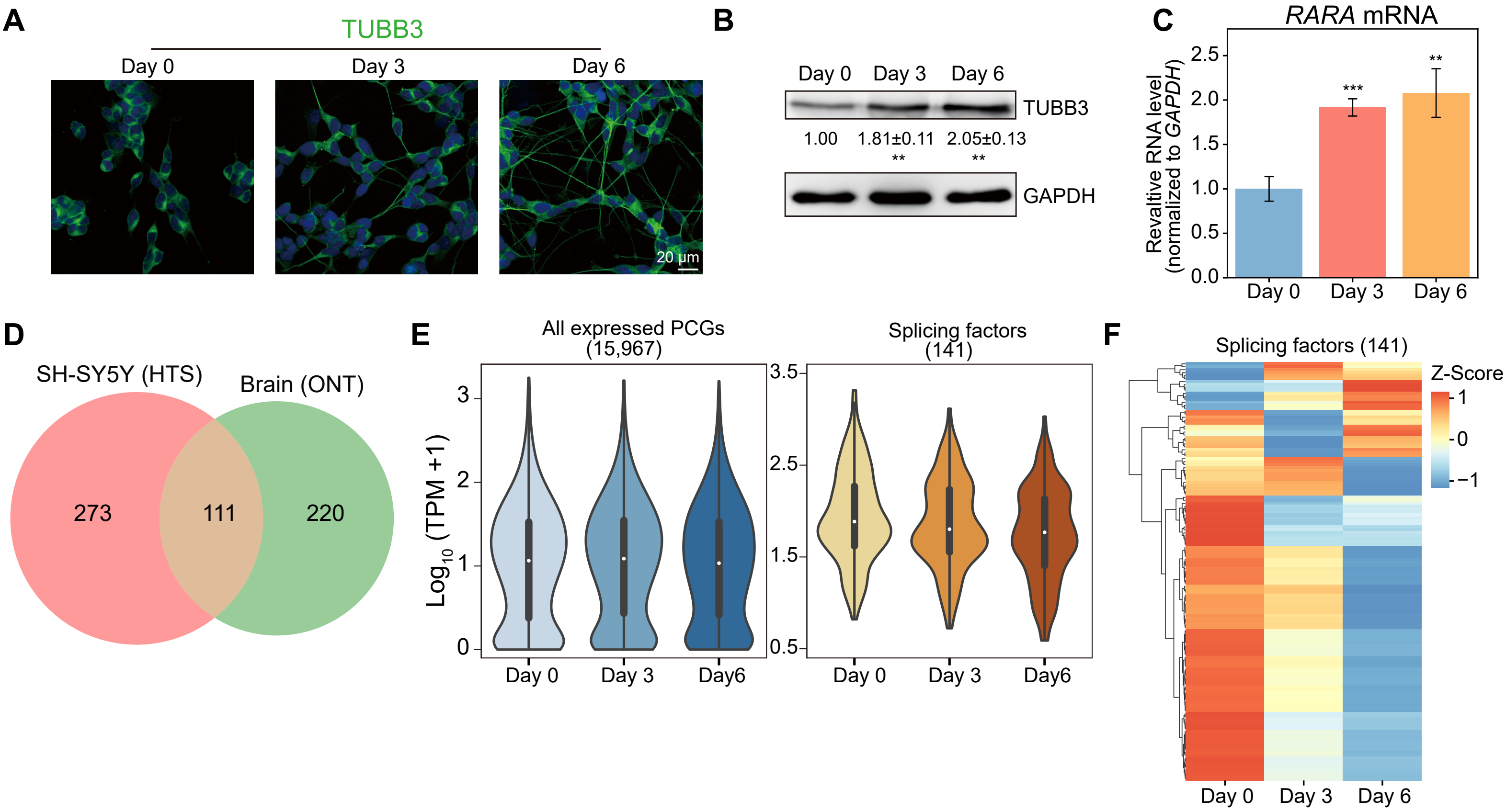

Supplemental Figure S12. Expression of splicing factors and EIciRNAs during neuronal differentiation.

A. Representative immunofluorescence (IF) images showing the distribution of the neuronal marker TUBB3 during RA-induced SH-SY5Y cell differentiation. **B**. Western blot of TUBB3 protein during RA-induced differentiation of SH-SY5Y cells. GAPDH was used as the loading control. **C**. RT-qPCR analysis of *RARA* mRNA levels during RA-induced differentiation of SH-SY5Y cells. Error bars represent SD in triplicate experiments and P-values were calculated with two-tailed Student's *t*-test. **$p < 0.01$; ***$p < 0.001$. **D**. Venn diagram showing the overlap of EIciRNAs detected from SH-SY5Y cells and published ONT data of the human brain (GSE141693). **E**. Violin plots showing expression levels of 15,967 protein-coding genes (TPM ≥ 1) and 141 splicing factors (Papasaikas et al. 2015) during the RA-induced differentiation of SH-SY5Y cells. **F**. Heatmap showing expression levels of 141 splicing factors in **E** during the RA-induced differentiation of SH-SY5Y cells.

Supplemental Figure S13

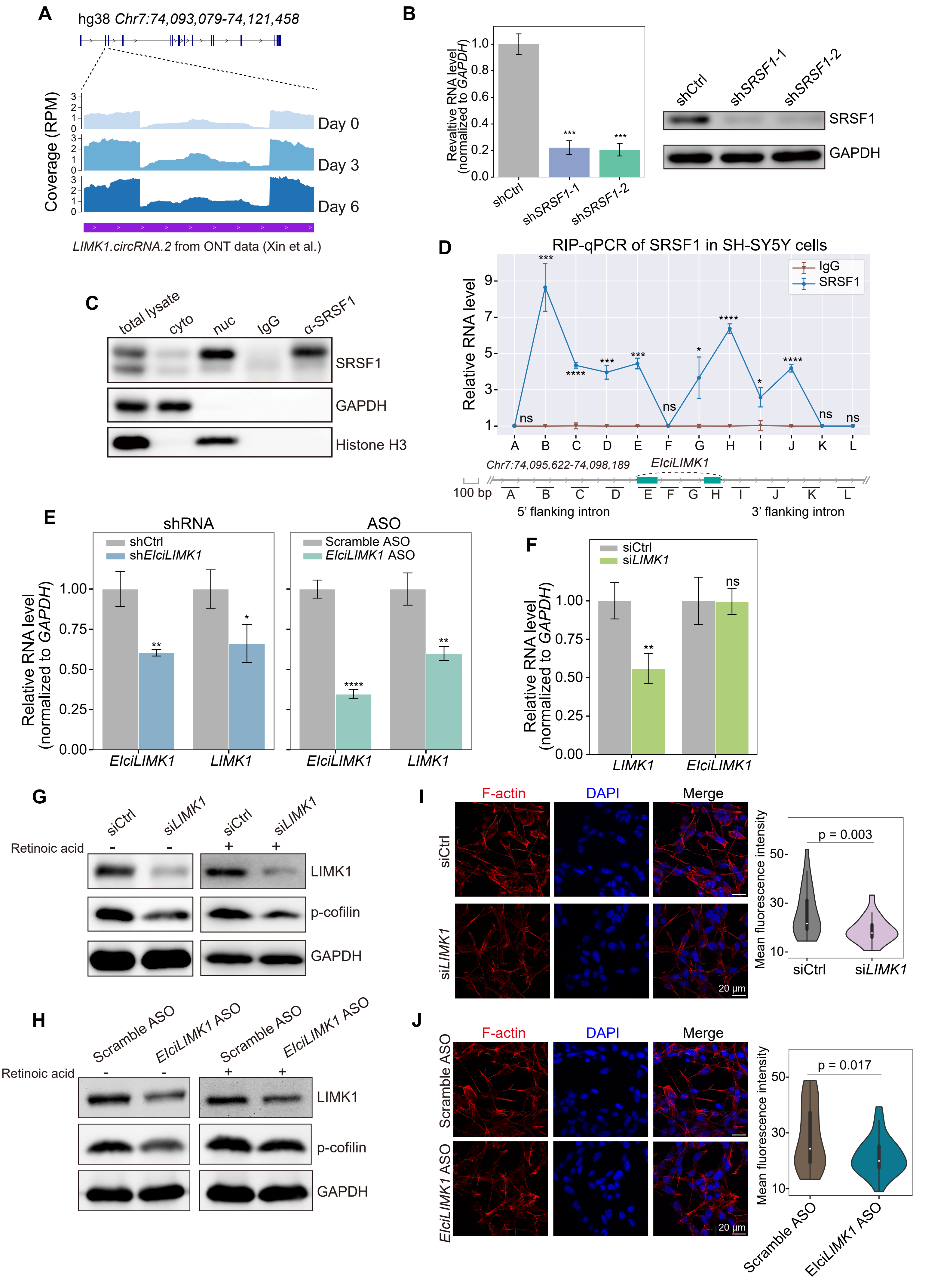

Supplemental Figure S13. *EIciLIMK1* promotes neuronal differentiation by enhancing *LIMK1* expression.

A. IGV snapshot showing *EIciLIMK1* signals during the RA-induced differentiation of SH SY5Y cells. *EIciLIMK1* was also confirmed by the published ONT data from the human brain (Xin et al. 2021). **B**. RT-qPCR and western blot showing the SRSF1 level in SH-SY5Y cells after *SRSF1* knockdown. shCtrl, shRNA with scrambled sequences; sh*SRSF1*-1 and sh*SRSF1*-2, two independent shRNAs against *SRSF1*. **C**. Western blot assessing the distribution of nuclear and cytosolic markers, as well as SRSF1, in whole cell lysate (total), cytosol (cyto), and nucleus (nuc) obtained through the nucleocytoplasmic separation assay. SRSF1 RNA immunoprecipitation (RIP) samples were also included in the analysis. **D**. RT-qPCR analysis of SRSF1 RIP samples showing the binding of SRSF1 on *EIciLIMK1* and the flanking introns in SH-SY5Y cells. The primers covering the *EIciLIMK1* locus were indicated below. **E**. RT-qPCR analysis of the expression levels of *EIciLIMK1* and *LIMK1* mRNA upon shRNA- or ASO-mediated knockdown of *EIciLIMK1* in SH-SY5Y cells. sh*EIciLIMK1* and *EIciLIMK1* ASO were targeting the BSJ sequence of *EIciLIMK1*. Scramble ASO, ASO with scramble sequences. **F**. RT-qPCR analysis of the expression levels of *LIMK1* mRNA and *EIciLIMK1* upon knockdown of *LIMK1* mRNA with siRNA. siCtrl, siRNA with scramble sequences; si*LIMK1*, siRNA against *LIMK1* mRNA. **G-H.** Western blot showing the expression levels of LIMK1 and p-cofilin protein after knockdown of *LIMK1* mRNA with siRNA (**G**), or knockdown of *EIciLIMK1* with ASO (**H**) in SH-SY5Y cells followed by uninduced or RA-induced differentiation. GAPDH was used as the loading control. **I-J.** Representative IF images showing the changes of F-actin after knockdown of *LIMK1* mRNA with siRNA (**I**), or knockdown of *EIciLIMK1* with ASO (**J**) in SH-SY5Y cells followed by RA-induced differentiation (left). Quantitative analysis of F-actin fluorescence intensity was shown (right). N=40. In B, and D-F, error bars represent SD in triplicate experiments and P-values were calculated with two-tailed Student's *t*-test. $^*p < 0.05$; $^{**}p < 0.01$; $^{***}p < 0.001$; $^{****}p < 0.0001$. In I and J, P-values were calculated with the Wilcoxon rank-sum test.

Descriptions of Supplemental Tables S1-S11

Supplemental Table S1.

A. circRNAs detected from RNase R-treated RNA-seq data of HEK293 cells using CIRI2.

B. EIciRNAs detected from RNase R-treated RNA-seq data of HEK293 cells using FEICP.

Supplemental Table S2.

A. Information of 20 EIciRNAs whose expression levels were determined using RT-qPCR to validate the accuracy of FEICP pipeline.

B. Information of 10 EIciRNAs detected by FEICP but missed out by isoCirc in HEK293 cells used for validation via RT-PCR.

Supplemental Table S3.

Intron retention in linear RNAs (LIR) detected from HEK293 cells using IRFinder (IRratio≥0.1).

Supplemental Table S4.

A. List of sequence features used for classification of three categories of introns using deep learning.

B. Top 50 sequence features distinguishing three categories of introns from each other.

Supplemental Table S5.

Counts of sgRNAs in CRISPR screening in P-In and E-In reporter cells.

Supplemental Table S6.

Expression levels of EIciRNAs upon *SRSF1* knockdown in HEK293 cells.

Supplemental Table S7.

A. Gene counts in HEK293 cells upon knockdown of *SRSF1*.

B. Differential gene expression analysis with DESeq2 upon *SRSF1* knockdown in HEK293 cells.

Supplemental Table S8.

Differential analysis of LIR in *SRSF1* knockdown HEK293 cells using IRFinder.

Supplemental Table S9.

Gene counts detected from RNA-seq data during SH-SY5Y differentiation.

Supplemental Table S10.

Public datasets used in this study.

Supplemental Table S11.

Oligonucleotide sequences used in this study.

Supplemental Methods

Analysis of gene expression from RNA-seq data

For all RNA-seq data analysis, STAR (Dobin et al. 2013) was used to map sequencing reads onto the reference genome (GRCh38 for human and GRCm38 for mouse) using the standard parameters used by ENCODE RNA-seq pipeline. RSEM v1.3.1 (Li and Dewey 2011) was used to estimate the abundance of all transcripts in GENCODE gene annotation GTF file (Frankish et al. 2021).

Differential expression analysis of genes

The read counts of genes were extracted from the RSEM results, and the counts for all genes in each sample were merged into one matrix. The expression matrix was used as the input of R package DESeq2 (Love et al. 2014) to assess the differential expression. A gene was considered significantly differentially expressed with the cutoff (fold change ≥ 1.5 or ≤ 0.667, and p-value < 0.05).

Definition of EcircRNAs

It was easier to confirm the existence of IR events than to confirm their non-existence, due to the possibility of IR detection failure from insufficient RNA sequencing depth. To minimize the false positive outcomes, a circRNA was considered as an EcircRNA only when it met the following criteria: (1) either its start or end coordinates matched to the exon boundary of known transcripts; (2) no EIciRNA with the same BSJ was detected; (3) the total number of BSJ supporting this circRNA in all samples must be at least 5.

Tissue specificity analysis of genes and circRNAs

Tau method (Yanai et al. 2005) was used to characterize the tissue specificity of genes and circRNAs. The tau value was calculated using the following formula:

$$\tau = \frac{\sum_{i=1}^{n}(1 - y_i)}{n - 1}; y_i = \frac{x_i}{\max\limits_{1 \leq i \leq n}(x_i)}$$

x_i indicated the expression level of molecule x in tissue i. Transcripts per million (TPM) was calculated to represent the expression levels of protein-coding genes (PCGs) or lncRNAs, and backspliced reads per million (BRPM) was calculated to represent the expression levels of circRNAs. The tissue specificity index τ values ranged from 0 (expressed in all tissues) to 1 (expressed exclusively in one tissue).

Correlations between expression levels of RBPs and EIciRNAs

Expression levels of RBPs (Gerstberger et al. 2014) were calculated across 19 human tissues. 1,031 RBPs with TPM ≥ 1 in at least one tissue were regarded as expressed, and selected for further analysis. 1,628 EIciRNAs whose total BRPM in 19 tissues was at least 0.1 were selected for further analysis. Spearman's correlation coefficients between expression levels of the RBPs and EIciRNAs were calculated, followed by hierarchical clustering using hclust function in R based on Euclidean distances and ward.D2 clustering method. Correlations between RBP and sum of EIciRNA BRPM in each tissue were computed, and then all RBPs were ranked according to the correlations. We selected the top 100 RBPs with the most negative correlations, and performed protein-protein interaction analysis using STRING (Szklarczyk et al. 2019), followed by clustering into 3 clusters using k-means clustering algorithm, and the top-ranked associated biological process was shown for each cluster.

The correlation between IR and gene expression

For the detection of EIciRNAs, 38 total RNA-seq data from 19 human tissues were analyzed using FEICP. As for the detection of IR in linear RNAs, all poly(A)-plus RNA-seq data of human tissues from ENCODE portal were downloaded, and those with any problem about the data quality were excluded, leaving 70 datasets from 31 tissues. IRFinder (Middleton et al. 2017) was then used to analyze these datasets, and introns with IRratio ≥ 0.1 were considered to be retained in linear RNAs. For each tissue, genes were binned into deciles according to their TPM values and the proportion of corresponding IR was calculated in each decile.

Defining of CIR, LIR, and NCI used for deep learning

For each intron, the number of reads for exon1-intron junction (E1I), intron-exon2 junction (IE2) and exon1-exon2 junction (E1E2) was calculated. The metric percent intron retention (PIR) was then calculated using the following formula as described in (Braunschweig et al. 2014):

$$PIR = \frac{\frac{E1I + IE2}{2}}{\frac{E1I + IE2}{2} + E1E2}$$

FEICP pipeline was used to detect introns retained in EIciRNAs from RNase R-treated RNA-seq data, and those introns with PIR ≥ 0.1 were selected as CIRs. Similarly, EcircRNAs from the same sequencing data were detected using CIRI2 and the introns with PIR ≤ 0.02 within EcircRNAs were selected as NCI. IRFinder was used to analyze poly(A)-plus RNA-seq data. Those introns with IRratio ≥ 0.1 without warnings were selected as LIRs.

Construction of the NNetwork for intron classification

The deep learning was performed as described with some modifications (Yeom et al. 2021). Introns belonging to only one group were selected, and their sequence features were calculated for deep learning. The sequence features were composed of five groups: sequence motifs, transcript features, RNA secondary structure, nucleosome positioning, and conservation. FIMO (Grant et al. 2011) was used for searching RBP binding motifs. RNAfold from ViennaRNA package (Lorenz et al. 2011) was used for the prediction of RNA secondary structure. NuPoP (Xi et al. 2010) was used for the prediction of nucleosome positioning, and MaxEntScan (Yeo and Burge 2004) was used for the prediction of splice site strength. For conservation analysis, conservation scores represented by PhastCons (Siepel et al. 2005) for multiple alignments of 99 vertebrate genomes to the human genome were downloaded from UCSC Genome Browser (https://hgdownload.cse.ucsc.edu/goldenPath/hg38/phyloP100way) (Kent et al. 2002) , and bwtool (Pohl and Beato 2014) was used to calculate the sequence conservation. All other sequence features were calculated using custom Python scripts with the help of BEDTools (Quinlan and Hall 2010). A three-layer deep neural network was constructed using Keras v2.6.0 (https://keras.io) which used TensorFlow (Abadi et al. 2016) as the backend, and was trained using a five-fold cross-validation fold method, to predict the group to which an intron belonged. For assessment of the DNN performance, the ROC curve was plotted, and AUC was calculated using the Python package scikit-learn v1.0.2 (Pedregosa et al. 2011). The decrease of the AUC value when the values of one feature were replaced by its median was used to represent the importance of this feature.

Analysis of RNA-binding protein (RBP) binding in CIR

Public RNA-seq data from K562 under accession SRR1049832 and SRR1049833 were re-analyzed with FEICP, followed by the identification of CIR and NCI. The peak bed files and signal bigwig files for 134 RBPs eCLIP-seq (Van Nostrand et al. 2020) in K562 cells were downloaded from the ENCODE data portal. For each RBP, BEDTools intersect was used to obtain the peaks located in introns. The peak signals were extracted using bwtool from the corresponding bigwig files. The signals of each RBP in CIR over NCI were compared and P-values were calculated with Wilcoxon rank-sum test.

Calculation of the proportion of CIR and genes generating EIciRNAs in human genome

EIciRNAs from 244 public RNA-seq data from 102 human tissue or cell samples were analyzed using FEICP. For n in any of 1, 2, …,102, n samples were randomly selected, and the total number of CIR was calculated from them. This process was iterated 1,000 times and then the mean of each number of samples was calculated. To fit these means, a function with two exponential terms, $f(x) = C_1(1\text{-}e^{-C_2 x})+C_3(1\text{-}e^{-C_4 x})$, was used. The limit of this function when the variable x was close to ∞, $C_1 + C_3$, was used to represent the total proportion of CIR in human genome. Calculation of the total proportion of protein-coding genes generating EIciRNAs was performed in a similar approach.

RNA-seq library preparation

Total RNA was extracted using TRIzol reagent (Invitrogen) according to the manufacturer's instructions, followed by DNase treatment. Next, DNase-treated RNA was used for library preparations with the NEBNext Ultra RNA Library Prep Kit for Illumina (NEB, USA) following manufacturer's recommendations. For circRNA sequencing, RNA was digested with 3 U of RNase R (Epicentre, USA) per µg of RNA to remove linear RNAs before library preparation. Each library was sequenced on a NovaSeq 6000 (Illumina) platform and 150-bp paired-end reads were generated, with a sequencing depth of ~30 million reads.

Cell culture, induction of differentiation, and transfection

Human embryonic kidney cells (HEK293) were cultured with DMEM supplemented with 10% FBS and 1% penicillin/streptomycin (P/S), incubated at 37°C, 5% CO2. SH-SY5Y cells were from Cell Bank/Stem Cell Bank, Chinese Academy of Sciences (SCSP-5014), cultured with DMEM supplemented with 12% FBS and 1% P/S at 37°C, 5% CO_2. For differentiation of SH-SY5Y, cells were seeded in a plate with a density of $1\times10^5/cm^2$ overnight and changed with the complete medium to differentiation medium (DMEM, 1% FBS, 1% P/S, 10 µM Retinoic Acid). Cells were cultured in the dark at the third and sixth day. The medium was changed every two days. For HEK293 cells, the plasmids (2 µg/ml, final concentration) were transfected to each well using Lipofectamine 2000 (Invitrogen, 11668019). For SH-SY5Y cells, the plasmids (5 µg/ml, final concentration) were transfected with Lipofectamine 3000 (Invitrogen, L3000008), and 100 nM (final concentration) siRNAs or 2-O-methyl RNA/DNA antisense oligonucleotides (ASOs) were transfected to each well with RNAiMax (Invitrogen, 13778075). All procedures were performed according to the manufacturer's protocol.

Whole-genome CRISPR knockout screen and data analysis

The human CRISPR knockout pooled library (Brunello) was purchased from Addgene (#73179) and infected into reporter cell lines, according to the instructions. After 48 h infection, the infected cells were selected with 1 µg/ml puromycin for seven days. The uninfected HEK293 cells were used to set the control gate of mCherry fluorescence, and mCherry-positive cells were subjected to sorting by GFP using FACS. Among the GFP-positive cells, those with the GFP intensity falling in the top or bottom 10% were collected separately, and then the genomic DNA of 4×10^6 cells was extracted. The sgRNAs were amplified using PCR, and the purified PCR product was subjected to HTS. Primers used for PCR amplification are listed in Supplemental Table S11. For CRISPR screen data analysis, adapters and sequencing primers were removed, and sgRNA sequences were extracted using a custom Python script from raw

fastq files. Then the sgRNA sequences were aligned to the Brunello library sequences using bowtie (Langmead et al. 2009). SAMtools idxstats (Li et al. 2009) was used to estimate read counts for each sgRNA from the alignment results. To assess the enrichment of sgRNAs between the input and High-GFP, or Low-GFP groups, we used the MAGeCK (v0.5.9.5) algorithm (Li et al. 2014) to determine positive enrichment scores for each gene via robust rank aggregation (RRA). Candidate genes were defined as effective sgRNAs ≥ 3, fold change ≥ 1.5, and P-value < 0.05.

Image processing and quantification

Images and the quantifications were analyzed by image processing software, Fiji (Schindelin et al. 2012). For semi-quantitative RT-PCR and western blot experiments, the images were rotated so that the bands were lined up horizontally. The density of the band presented inside the “mountains” was measured and analyzed. The background was subtracted from an area above each band that was the same size as the respective band. For IF images, the color channels were split and merged with Fiji. The fluorescence was analyzed and measured. The cell regions were defined by freehand.

Nuclear run-on and sequencing

Nuclear run-on was performed as described in the previous study (Li et al. 2015; Core et al. 2008). Briefly, the cells were harvested in ice-cold hypotonic solution (10 mM Tris-HCl pH 7.4, 150 mM KCl, 4 mM MgOAc, 200 units/ml RNase Inhibitor (ABclonal, RK21401)) and were centrifuged at 1,000 g for 3 min at 4°C. The pellets were resuspended in lysis buffer (10 mM Tris-HCl pH 7.4, 150 mM KCl, 4 mM MgOAc, 0.5% NP-40, 10% glycerol, 200 units/ml RNase Inhibitor) and centrifugated by sucrose density gradient. The pellets were lysed with run-on buffer (10 mM Tris-HCl pH 7.4, 5 mM $MgCl_2$, 150 mM KCl, 1% sarkosyl, 2 mM DTT) and added with 10 mM ATP, CTP, GTP, BrUTP, and 200 units/ml RNase inhibitor. The mixtures were incubated for 5 min at 30°C. The RNA was extracted by TRIzol reagent (Invitrogen) and treated with DNase I (Thermo Fisher, EN0521) digestion. For global run-on sequencing (GRO-seq), the RNA was fragmented by Magnesium and then anti-BrdU antibody (NOVUS, NB500-235) was used to immunoprecipitate the fragmented RNA. The library was constructed following “RNA-seq library preparation”. For real-time quantitative PCR (RT-qPCR) assays, anti-BrdU antibody was used to immunoprecipitate the nascent RNA, which was reverse transcribed to cDNA. RT-qPCR primers used are listed in Supplemental Table S11.

Data analysis of GRO-seq

Raw sequence reads were trimmed for adaptor using cutadapt (Martin 2011). Trimmed reads were mapped to GRCh38 using bowtie2 (Langmead and Salzberg 2012) and only uniquely mapped reads were retained. Density of GRO-seq was then calculated for plus and minus strands and normalized using the total uniquely mapped reads using bamCoverage in deepTools (Ramírez et al. 2016). BEDTools was then used to calculate the signal density of GRO-seq on genes.

Prediction of full-length of EcircRNAs from RNA-seq data

Psirc (pseudo-alignment identification of circular RNAs) v1.0 (Yu et al. 2021) was used to predict the full-length of EcircRNAs from RNA-seq data according to the authors’ instructions (https://github.com/Christina-hshi/psirc).

Calculation of *Alu* counts in introns

To determine the number of *Alu* in flanking introns of circRNAs, flanking introns of circRNAs were extracted and then *Alu* was counted using BEDTools. The *Alu* elements were extracted from the repeatMasker file downloaded from UCSC genome Browser (http://hgdownload.soe.ucsc.edu/goldenPath/hg38/database/rmsk.txt.gz).

Gene enrichment analysis

Gene ontology (GO) analysis was performed using the R package ClusterProfiler v4.7.1.003 (Wu et al. 2021).

EIciRNA detection from ONT data

isoCirc v1.0.4 was used to re-analyze the nanopore sequencing data of HEK293 and 12 human tissues generated from the previous report (Xin et al. 2021) to obtain the full-length of circRNAs. Then their host transcripts were identified as in "The FEICP pipeline" section. Those circRNAs that retained the whole intron of the corresponding transcript were regarded as EIciRNAs.

Detection of EIciRNAs using CIRI-full and CYCLeR

CIRI-full (Zheng et al. 2019) was performed on four replicates of RNase R-treated RNA-seq data in HEK293 cells from this study, according to the instructions (https://ciri-cookbook.readthedocs.io/en/latest/CIRI-full.html). Briefly, CIRI-full Pipeline module was used to automatically detect and reconstruct circRNAs, followed by estimating the related abundance of isoforms according to the output of CIRI-full using CIRI-vis. CYCLeR (Stefanov and Meyer 2023) was performed on the same set of RNA-seq data according to the instructions (https://raw.githubusercontent.com/stiv1n/CYCLeR/main/CYCLeR_workflow.pdf), in which public RNA-seq data (SRR22315104, SRR22315105, SRR22315106, and SRR22315107) without RNase R-treatment was used as control to identify circRNA specific features. For both software, isoforms with a whole intron retained in circRNAs were considered as EIciRNAs.

Detection of cryptic exons included in circRNAs

The full-length circRNAs in HEK293 cells were detected from the ONT data generated by Xin et al. using isoCirc (Xin et al. 2021). Exons met: (1) not annotated as exons in gene annotation file; (2) overlapped with introns, were extracted using BEDTools and considered as cryptic exons included in circRNAs.

Analysis of RBP binding density on EIciRNAs

Overlap of the 1,031 RBPs contained in Fig. 2E with the ENCODE eCLIP-seq of K562 resulted in 98 RBPs, in which 80 and 18 belonged to "Cluster A" and "Cluster B", respectively. The binding density on retained introns, circular exons, and flanking introns of 1,231 EIciRNAs in "Cluster 1" was calculated using deepTools for each RBP. The mean binding density on these regions was calculated and compared for RBPs in "Cluster A" and "Cluster B", respectively.

Calculation of proportions of genes generating EIciRNAs or EcircRNAs

EIciRNAs were detected from 38 total RNA-seq data of 19 human tissues as described in "The correlation between IR and gene expression" section, and EcircRNAs were detected from the same sets of RNA-seq data using CIRI2. Genes were binned into deciles according to expression levels and proportions of genes generating EIciRNAs or EcircRNAs were calculated for each bin in each tissue.

Definition of EIciRNA-match genes

All expressed protein-coding genes (TPM$\geq$1) were sorted according to TPM. For each EIciRNA, the gene with the closest expression level to its parental gene was referred as its "EIciRNA-match" gene.

Construction of neural network for predicting intron retention in linear RNAs

Based on the results of IRFinder, introns with IRratio $\leq$ 0.02 were considered as not retained in linear RNAs. The 1,309 sequence features of these spliced introns and LIR were subjected to

train a three-layer neural network tasked with predicting whether an intron was retained in linear RNAs or not (NNetwork-linear). The construction of NNetwork-linear and the its application in CIR were similar to description in "Construction of the NNetwork for intron classification" section.

Assessment of enrichment of isoCirc-detected circRNAs

The full-length sequence of EIciRNAs was detected from isoCirc, followed by adding the first 30 nucleotides of EIciRNAs and an additional 150 Ns to the end of EIciRNAs. Kallisto (Bray et al. 2016) was used to quantify abundance of these transcripts from the RNase R-treated RNA-seq data generated in this study and the public total RNA-seq data without RNase R treatment (SRR22315104, SRR22315105, SRR22315106, and SRR22315107). P-values were calculated between RNase R+ and RNase R- samples using Student's *t*-test. EIciRNAs with FoldChange ≥ 1.5 and P-value < 0.05 were considered to be enriched by RNase R-treatment.

Assessment of performance of FEICP on sequencing reads with different fragment size and read length

The paired-end 250 (PE250) RNA-seq data without fragmentation was downloaded from NCBI (SRA: SRR7350933), and FEICP was applied to this dataset to detect EIciRNAs. BSJ reads of these EIciRNAs were extracted, followed by inferring their fragment length based on the alignment file and exon-intron structure of EIciRNAs. BSJ reads were categorized into five groups based on their fragment size: 250-350, 350-400, 400-450, 450-500, and ≥500. FEICP was also used to detect EIciRNAs from sequencing reads with varied lengths (PE50, PE100, PE150, PE200) obtained by trimming PE250 data. For each fragment group and read length group, the number and length of EIciRNAs were counted.

Metaplot

To visualize the signal density of DNase-seq and ChIP-seq, the corresponding bigwig files, which represented the fold change of signal over control from ENCODE data portal were downloaded (The ENCODE Project Consortium 2012). The average signal density around the transcript start site (TSS) of genes was then calculated using computeMatrix reference-point implemented in deepTools. For TT-seq of four cell lines, the average signal density along the whole gene body was calculated using computeMatrix scale-regions implemented in deepTools.

t-SNE algorithm

For dimensional reduction of sequence features of introns, R package Rtsne v0.16 (https://github.com/jkrijthe/Rtsne) was used to perform t-SNE algorithm (van der Maaten and Hinton 2008; van der Maaten 2014).

Public RNA-seq data for verifying sequence features identified by deep learning

In Supplemental Fig. S8, we used publicly available RNase R-treated RNA-seq data for the identification of CIR and NCI. We used polyA-plus RNA-seq data for the identification of LIR. For CIR and NCI, RNA-seq data under bioproject PRJNA722575 for HEK293, HeLa and SH-SY5Y (Liu et al. 2021), and accession SRR1049832, SRR1049833 for K562 were downloaded. As for LIR, data for HEK293, K562 and SH-SY5Y cells were downloaded from the RNA atlas, which provides a comprehensive transcriptome atlas of 300 human tissues and cell lines, and under accession SRR4035637 from our previous study for HeLa cells.

Expression analysis of EIciRNAs after *SRSF1* knockdown

Considering *SRSF1* knockdown could have effect on both linear and back splicing, the following method was used to normalize the read counts of EIciRNAs from FEICP. First, the back-spliced junction counts from CIRI2 and forward-spliced junction counts from STAR were combined into a single matrix of spliced junctions, which was then used to calculate the normalization factors among different biosamples using the generalized linear model (GLM)

implemented in estimateSizeFactors function in DESeq2. Second, read counts of EIciRNAs from FEICP were normalized through dividing BSJ counts by the corresponding normalization factors. EIciRNAs with fold change ≥ 2 or fold change ≤ 0.5 were selected as differentially expressed EIciRNAs.

Analysis of SRSF1 iCLIP-seq data in HEK293 cells

The SRSF1 iCLIP-seq data in HEK293 was downloaded under accession SRR3734557, SRR3734558 and SRR3734559. Data analysis of iCLIP-seq data was performed with the previously described pipelines with some modifications (Howard et al. 2018; Wang et al. 2021). Briefly, the first 9 bp in each read (the 5-9 bp of random nucleotides was used for identifying PCR duplicates) were removed, followed by trimming off the adaptor sequence. Trimmed reads were then aligned to the human genome (hg38) allowing no more than one alignment using STAR (--outFilterMultimapNmax 1). Reads were then truncated to their 5′ ends, whose genomic locations were considered as the crosslinking sites. Those reads with the same genomic location and 5-bp random nucleotide were duplicated. For motif analysis, a 41-nt region was created by extending 20-nt upstream and downstream of each crosslinking site, followed by extraction of sequences. These sequences were subjected to AME (McLeay and Bailey 2010) for searching motifs, and the outputted motifs were compared against the Ray2013 Homo sapiens database (Ray et al. 2013), including 102 known RNA motifs of 80 RBPs, using TOMTOM (Tanaka et al. 2011). The 6-nt motif matched to the known SRSF1-binding motif with p-value < 0.05 was selected.

Plasmids and construction of plasmids

All plasmids were constructed through restriction enzyme digestion along with ligation or recombination (Vazyme). The intron from *EIciPAIP2* or *EIciEIF3J*, including 10-nt exon sequences by the exon-intron boundary was inserted in circGFP plasmid (a gift from Z. Wang) using EcoRI and SalI restriction sites. To knock down *SRSF1* and *EIciLIMK1*, the target sequences of *SRSF1* and *EIciLIMK1* were cloned into pLKO.1 vector between AgeI and EcoRI sites. The shRNA plasmids were used to knockdown *DHX9* (sh*DHX9*, TRCN0000001208), *DHX15* (sh*DHX15*, TRCN0000000006), *HNRNPC* (sh*HNRNPC*, TRCN0000006645), *LSM6* (sh*LSM6*, TRCN0000074718), *SNRPA1* (sh*SNRPA1*, TRCN0000072503), *TGS1* (sh*TGS1*, TRCN0000060829), *PABPC1* (sh*PABPC1*, TRCN0000074640) was obtained from the MISSION shRNA Library (Sigma-Aldrich, Germany). The negative control (shC002) for shRNA plasmid was purchased from Sigma-Aldrich. The coding sequences (CDS) of *SRSF1* and *LIMK1* were cloned in p3×FLAG-Myc-CMV-24, respectively. The plasmid of *EIciLIMK1* overexpression was constructed with *EIciLIMK1* corresponding sequences plus the 1.0 kb from upstream and downstream of flanking sequence. PCR primers are listed in Supplemental Table S11.

Construction of the reporter cell lines for CRISPR screening

The cell lines stably expressing GFP and mCherry were constructed as follows. (1) Two GFP-expressing plasmids and a mCherry-expressing plasmid were simultaneously transfected into HEK293 cells; (2) After 48 h transfection, the cells were treated with 600 μg/ml geneticin (G418) for selection in the following seven days; (3) The single cells co-expressing GFP and mCherry fluorescence were sorted by FACS and then seeded to the 96-well plate; (4) Subsequently, the cells were cultured and subjected to 300 μg/ml G418 selection for ten days. The single-clone highly co-expressing GFP and mCherry fluorescence was re-sorted to single cell and seeded to the next the 96-well plate. The step (4) was repeated. Finally, the single clone with more than 97% co-expressing GFP and mCherry fluorescence cells was selected and scaled up as the reporter cell lines.

Fluorescence-activated cell sorting (FACS) and flow cytometry

For detecting GFP and mCherry fluorescence from reporter cell lines, the cells were first detached with trypsin-EDTA and resuspended in sorting buffer (1% FBS, 1 mM EDTA in PBS).

The 488 nm excitation was used to detect the fluorescence of GFP, and 561 nm excitation was used to detect the fluorescence of mCherry. The MoFlo Astrios Cell Sorter platform (Beckman Coulter) was used to sort single cells co-expressing GFP and mCherry, and distribute the cells into a 96-well plate pre-filled and pre-warmed with 150 µl DMEM supplemented with 10% FBS and 1% P/S. For measurement of GFP intensity in *SRSF1* knockdown reporter cells, the CytoFLEX platform (Beckman Coulter) was used. The FACS data was analyzed with FlowJo.

Western blotting

Whole-cell lysates were lysed in RIPA lysis buffer (50 mM Tris-HCl pH 8.0, 150 mM NaCl, 5 mM EDTA, 1% NP-40, 0.1% SDS, 1× Protease Inhibitor Cocktail (TransGen, DI101-01), 1× Phosphatase Inhibitor Cocktail (TransGen, DI201-01)) and quantified using Bicinchoninic Acid methods (BCA). The proteins were separated on SDS-PAGE gels and transferred to nitrocellulose membranes (Millipore). The ECL western blotting procedure was used for HRP detection (GE Healthcare). Fiji was used to quantify the bands. These antibodies were utilized for western blots: anti-SRSF1 (Santa Cruz, #sc-33652), anti-LIMK1 (CST, #3842), anti-Phospho-Cofilin (Ser3) (Proteintech, 29715-1-AP), anti-GAPDH (Proteintech, 10494-1-AP), anti-Histone3 (Signalway, #21137), anti-TUBB3 (Proteintech, 66375-1-Ig), anti-GFP (TransGen, HT801-01).

Immunofluorescence (IF) staining

Cells were plated on poly-D-lysine coated coverslips. The plated cells were washed twice with PBS and then fixed with 4% paraformaldehyde (PFA, methanol-free) for 10 min at room temperature, following by washing three times with PBST and permeabilized with PBS plus 0.5% Triton X-100 for 10 min on ice. Then the cells were blocked with blocking buffer (PBST plus 1% BSA) for 30 min at room temperature. For the microtube, the plated cells were incubated with anti-TUBB3 (Proteintech, 66375-1-Ig) diluted in blocking buffer at 4℃ overnight. After washing three times with PBST, the plated cells were incubated with secondary antibodies diluted in blocking buffer for 2 h at room temperature in the dark. For F-actin, the plated cells were incubated with Actin-Tracker (Beyotime, C2205S) diluted in blocking buffer for 2 h at room temperature. Next, DAPI (Sigma-Aldrich, F6057) was used to stain nuclei. The images were taken at 25× (microtube) or 40× (F-actin) objective with ZEISS LSM 980 confocal microscope. Fiji was used to quantify the mean intensity of cells by freehand and forty cells were counted in each condition.

PCR reactions

The total RNA was extracted with TRIzol reagent (Invitrogen), and DNase I was used to digest the genomic DNA, according to the manufacturer's protocol. The cDNA was synthesized from the RNAs using reverse transcriptase (ABclonal, RK20400) in the presence of oligo dT or random hexamer primers. The RT-qPCR was performed with Universal SYBR Green Fast qPCR Mix (ABclonal, RK21204) on QuantStudio 3 real-time PCR Instrument according to recommended procedures. For semi-quantitative RT-PCR reaction, 13-25 cycles were used to amplify the segment of DNA. PCR and RT-qPCR primer sequences are listed in Supplemental Table S11.

EU labeling and purification of nascent RNA

The nascent RNA experiment was performed as described in the previous publication with minor modifications (Bao et al. 2018). In brief, the cells were incubated with 250 µM EU (RiboBio, C00064) for 2 h. The labeled cells were fixed with 90% ethanol for 30 min on ice and permeabilized with 0.5% Triton X-100 diluted in PBS for 15 min on ice. The click chemistry buffer (0.25 mM biotin-azide, 0.3 mM CuSO4, 0.6 mM THPTA, 1 mM aminoguanidine, 5 mM sodium L-ascorbate) was added in cells to link EU and biotin for 3 min at room temperature. The reaction was stopped by washing three times with stop buffer (0.5% Triton X-100, 2 mM EDTA) at room temperature. The cells were lysed with lysis buffer (20 mM Tris-HCl pH 7.4, 1 mM EDTA pH 8.0, 500 mM LiCl, 0.5% LDS, 5 mM DTT) and

sonicated in Bioruptor for 10 min on ice. The supernatant was incubated with 100 μl streptavidin-conjugated magnetic beads to capture biotinylated EU-labeled RNAs for 4 h at 4°C. After stringent washings, the RNA was eluted by elution buffer (10 mM EDTA pH 8.2, 95% formamide) for 5 min at 90°C and extracted by TRIzol reagent. All buffers were supplemented with 200 units/ml RNase inhibitor (ABclonal, RK21401).

Nucleocytoplasmic separation and RIP-qPCR

The cultured cells were rinsed twice with PBS and exposed to a UV cross-linker for 2 min at 254 nm and 400 mJ/cm^2. The irradiated cells were harvested in ice-cold lysis buffer (10 mM Tris-HCl pH 8.4, 140 mM NaCl, 1.5 mM $MgCl_2$, 0.5% NP-40) and centrifuged at 1,000 g for 3 min at 4°C. The supernatant was collected for WB and RNA as the fraction of cytoplasmic. The pellets were resuspended with ice-cold lysis buffer supplementary with 1/10th volume detergent stock (3.3% (w/v) Sodium Deoxycholate, 6.6% (v/v) Tween 40) and incubated on ice for 5 min. The nuclei were collected by centrifugation at 1,000 g for 3 min at 4°C. The nuclei were washed twice using ice-cold lysis buffer. The nuclei were lysed in RIPA buffer (50 mM Tris-HCl pH 8.0, 150 mM NaCl, 5 mM EDTA, 1% NP-40, 0.1% SDS) and sonicated in Bioruptor for 10 min on ice and then centrifuged at 12,000 g for 10 min at 4°C. The supernatant was pre-cleared for 1 h at 4°C with Protein G Magnetic Beads (Thermo). 50 μl beads was added with 5 μg anti-SRSF1 antibody (Santa Cruz, #sc-33652) or IgG (as control) together and incubated for 30 min at room temperature. The beads-antibody mixtures were washed twice using RIPA buffer and pre-cleared supernatant was added and incubated overnight at 4°C. The complex was washed five times with RIPA buffer, and three-tenths samples were saved as western blots. Samples were then extracted as RNA by TRIzol reagent (Invitrogen). All buffers were supplemented with RNase inhibitor and 1× protease-inhibitor cocktail (Sangon) freshly.

Data visualization

Circos plot was generated using the R package circlize (v0.4.15) (Krzywinski et al. 2009; Gu et al. 2014) to display the distribution of EIciRNAs in the human genome (hg38). For visualization of the overlap of the data set, the Python package matplotlib-venn v0.11.6 (https://github.com/konstantint/matplotlib-venn) was used to generate Venn diagrams and the R package ComplexUpset (v1.3.5) (https://github.com/krassowski/complex-upset/) was used to generate UpSet plots. The heatmap showing hierarchical clustering of correlations between expression levels of EIciRNAs and RBPs was generated with the R package ComplexHeatmap (v2.14.0) (Gu et al. 2016). Other heatmaps were plotted using the function clustermap in the Python package seaborn v0.11.1 (Waskom 2021). Read coverage of SRSF1 iCLIP-seq was calculated with bamCoverage in deepTools and visualized in IGV (Thorvaldsdóttir et al. 2013). All other plots in high-throughput data analysis were generated using the Python package matplotlib v3.5.3 (Hunter 2007) and seaborn v0.11.1, or the R package ggplot2 (v3.4.2) (Wickham 2016).

References for Supplemental Materials

Abadi M, Agarwal A, Barham P, Brevdo E, Chen Z, Citro C, Corrado GS, Davis A, Dean J, Devin M, et al. 2016. TensorFlow: large-scale machine learning on heterogeneous systems. *arXiv*:1603.04467 [cs.DC].

Bao X, Guo X, Yin M, Tariq M, Lai Y, Kanwal S, Zhou J, Li N, Lv Y, Pulido-Quetglas C et al. 2018. Capturing the interactome of newly transcribed RNA. *Nat Methods* **15**: 213-220.

Braunschweig U, Barbosa-Morais NL, Pan Q, Nachman EN, Alipanahi B, Gonatopoulos-Pournatzis T, Frey B, Irimia M, Blencowe BJ. 2014. Widespread intron retention in mammals functionally tunes transcriptomes. *Genome Res* **24**: 1774-1786.

Bray NL, Pimentel H, Melsted P, Pachter L. 2016. Near-optimal probabilistic RNA-seq quantification. *Nat Biotechnol* **34**: 525-527.

Core LJ, Waterfall JJ, Lis JT. 2008. Nascent RNA sequencing reveals widespread pausing and divergent initiation at human promoters. *Science* **322**: 1845-1848.

Dobin A, Davis CA, Schlesinger F, Drenkow J, Zaleski C, Jha S, Batut P, Chaisson M, Gingeras TR. 2013. STAR: ultrafast universal RNA-seq aligner. *Bioinformatics* **29**: 15-21.

The ENCODE Project Consortium. 2012. An integrated encyclopedia of DNA elements in the human genome. *Nature* **489**: 57-74.

Frankish A, Diekhans M, Jungreis I, Lagarde J, Loveland JE, Mudge JM, Sisu C, Wright JC, Armstrong J, Barnes I et al. 2021. GENCODE 2021. *Nucleic Acids Res* **49**: D916-D923.

Gerstberger S, Hafner M, Tuschl T. 2014. A census of human RNA-binding proteins. *Nat Rev Genet* **15**: 829-845.

Grant CE, Bailey TL, Noble WS. 2011. FIMO: scanning for occurrences of a given motif. *Bioinformatics* **27**: 1017-1018.

Gu Z, Eils R, Schlesner M. 2016. Complex heatmaps reveal patterns and correlations in multidimensional genomic data. *Bioinformatics* **32**: 2847-2849.

Gu Z, Gu L, Eils R, Schlesner M, Brors B. 2014. circlize Implements and enhances circular visualization in R. *Bioinformatics*. **30**: 2811-2.

Howard JM, Lin H, Wallace AJ, Kim G, Draper JM, Haeussler M, Katzman S, Toloue M, Liu Y, Sanford JR. 2018. HNRNPA1 promotes recognition of splice site decoys by U2AF2 in vivo. *Genome Res* **28**: 689-698.

Hunter JD. 2007. Matplotlib: a 2D graphics environment. *Comput Sci Eng* **9**: 90–95. doi:10.1109/MCSE.2007.55

Kent WJ, Sugnet CW, Furey TS, Roskin KM, Pringle TH, Zahler AM, Haussler D. 2002. The human genome browser at UCSC. *Genome Res* **12**: 996-1006.

Krzywinski M, Schein J, Birol I, Connors J, Gascoyne R, Horsman D, Jones SJ, Marra MA. 2009. Circos: an information aesthetic for comparative genomics. *Genome Res* **19**: 1639-1645.

Langmead B, Salzberg SL. 2012. Fast gapped-read alignment with Bowtie 2. *Nature methods* **9**: 357-359.

Langmead B, Trapnell C, Pop M, Salzberg SL. 2009. Ultrafast and memory-efficient alignment of short DNA sequences to the human genome. *Genome Biol* **10**: R25.

Li B, Dewey CN. 2011. RSEM: accurate transcript quantification from RNA-Seq data with or without a reference genome. *BMC Bioinformatics* **12**: 323.

Li H, Handsaker B, Wysoker A, Fennell T, Ruan J, Homer N, Marth G, Abecasis G, Durbin R, Subgroup GPDP. 2009. The sequence alignment/map format and SAMtools. *Bioinformatics* **25**: 2078-2079.

Li W, Xu H, Xiao T, Cong L, Love MI, Zhang F, Irizarry RA, Liu JS, Brown M, Liu XS. 2014. MAGeCK enables robust identification of essential genes from genome-scale CRISPR/Cas9 knockout screens. *Genome Biol* **15**: 554.

Li Z, Huang C, Bao C, Chen L, Lin M, Wang X, Zhong G, Yu B, Hu W, Dai L et al. 2015. Exon-intron circular RNAs regulate transcription in the nucleus. *Nat Struct Mol Biol* **22**: 256-264.

Liu Z, Tao C, Li S, Du M, Bai Y, Hu X, Li Y, Chen J, Yang E. 2021. circFL-seq reveals full-length circular RNAs with rolling circular reverse transcription and nanopore sequencing. *Elife* 10: e69457. doi: 10.7554/eLife.69457.

Lorenz R, Bernhart SH, Höner Zu Siederdissen C, Tafer H, Flamm C, Stadler PF, Hofacker IL. 2011. ViennaRNA Package 2.0. *Algorithms Mol Biol* **6**: 26.

Love MI, Huber W, Anders S. 2014. Moderated estimation of fold change and dispersion for RNA-seq data with DESeq2. *Genome Biol* **15**: 550.

Martin M. 2011. Cutadapt removes adapter sequences from high-throughput sequencing reads. *EMBnet journal* **17**: 10-12.

McLeay RC, Bailey TL. 2010. Motif Enrichment Analysis: a unified framework and an evaluation on ChIP data. *BMC Bioinformatics* **11**: 165.

Middleton R, Gao D, Thomas A, Singh B, Au A, Wong JJ, Bomane A, Cosson B, Eyras E, Rasko JE et al. 2017. IRFinder: assessing the impact of intron retention on mammalian gene expression. *Genome Biol* **18**: 51.

Papasaikas P, Tejedor JR, Vigevani L, Valcárcel J. 2015. Functional splicing network reveals extensive regulatory potential of the core spliceosomal machinery. *Mol Cell* **57**: 7-22.

Pedregosa F, Varoquaux G, Gramfort A, Michel V, Thirion B, Grisel O, Blondel M, Prettenhofer P, Weiss R, Dubourg V. 2011. Scikit-learn: Machine learning in Python. *J Mach Learn Res.* **12**: 2825-2830.

Pohl A, Beato M. 2014. bwtool: a tool for bigWig files. *Bioinformatics* **30**: 1618-1619.

Quinlan AR, Hall IM. 2010. BEDTools: a flexible suite of utilities for comparing genomic features. *Bioinformatics* **26**: 841-842.

Ramírez F, Ryan DP, Grüning B, Bhardwaj V, Kilpert F, Richter AS, Heyne S, Dündar F, Manke T. 2016. deepTools2: a next generation web server for deep-sequencing data analysis. *Nucleic Acids Res* **44**: W160.

Ray D, Kazan H, Cook KB, Weirauch MT, Najafabadi HS, Li X, Gueroussov S, Albu M, Zheng H, Yang A et al. 2013. A compendium of RNA-binding motifs for decoding gene regulation. *Nature* **499**: 172-177.

Schindelin J, Arganda-Carreras I, Frise E, Kaynig V, Longair M, Pietzsch T, Preibisch S, Rueden C, Saalfeld S, Schmid B et al. 2012. Fiji: an open-source platform for biological-image analysis. *Nat Methods* **9**: 676-682.

Siepel A, Bejerano G, Pedersen JS, Hinrichs AS, Hou M, Rosenbloom K, Clawson H, Spieth J, Hillier LW, Richards S et al. 2005. Evolutionarily conserved elements in vertebrate, insect, worm, and yeast genomes. *Genome Res* **15**: 1034-1050.

Stefanov SR, Meyer IM. 2023. CYCLeR-a novel tool for the full isoform assembly and

quantification of circRNAs. *Nucleic Acids Res* **51**: e10.

Szklarczyk D, Gable AL, Lyon D, Junge A, Wyder S, Huerta-Cepas J, Simonovic M, Doncheva NT, Morris JH, Bork P et al. 2019. STRING v11: protein-protein association networks with increased coverage, supporting functional discovery in genome-wide experimental datasets. *Nucleic Acids Res* **47**: D607-D613.

Tanaka E, Bailey T, Grant CE, Noble WS, Keich U. 2011. Improved similarity scores for comparing motifs. *Bioinformatics* **27**: 1603-1609.

Thorvaldsdóttir H, Robinson JT, Mesirov JP. 2013. Integrative Genomics Viewer (IGV): high-performance genomics data visualization and exploration. *Briefings in bioinformatics* **14**: 178-192.

van der Maaten L. 2014. Accelerating t-SNE using tree-based algorithms. *J Mach Learn Res* **2014**: 3221–3245.

van der Maaten L, Hinton G. 2008. Visualizing high-dimensional data using t-SNE. *J Mach Learn Res* **9**: 2579–2605.

Van Nostrand EL, Freese P, Pratt GA, Wang X, Wei X, Xiao R, Blue SM, Chen JY, Cody NAL, Dominguez D et al. 2020. A large-scale binding and functional map of human RNA-binding proteins. *Nature* **583**: 711-719.

Wang X, Li J, Bian X, Wu C, Hua J, Chang S, Yu T, Li H, Li Y, Hu S et al. 2021. CircURI1 interacts with hnRNPM to inhibit metastasis by modulating alternative splicing in gastric cancer. *Proc Natl Acad Sci U S A* **118**: e2012881118.

Waskom ML. 2021. Seaborn: statistical data visualization. *J Open Source Softw* **6**: 3021.

Wickham H. 2016. *ggplot2: elegant graphics for data analysis*. Springer-Verlag, New York. https://ggplot2.tidyverse.org.

Wu T, Hu E, Xu S, Chen M, Guo P, Dai Z, Feng T, Zhou L, Tang W, Zhan L et al. 2021. clusterProfiler 4.0: A universal enrichment tool for interpreting omics data. *Innovation (Camb)* **2**: 100141.

Xi L, Fondufe-Mittendorf Y, Xia L, Flatow J, Widom J, Wang JP. 2010. Predicting nucleosome positioning using a duration Hidden Markov Model. *BMC Bioinformatics* **11**: 346.

Xin R, Gao Y, Gao Y, Wang R, Kadash-Edmondson KE, Liu B, Wang Y, Lin L, Xing Y. 2021. isoCirc catalogs full-length circular RNA isoforms in human transcriptomes. *Nat Commun* **12**: 266.

Yanai I, Benjamin H, Shmoish M, Chalifa-Caspi V, Shklar M, Ophir R, Bar-Even A, Horn-Saban S, Safran M, Domany E et al. 2005. Genome-wide midrange transcription profiles reveal expression level relationships in human tissue specification. *Bioinformatics* **21**: 650-659.

Yeo G, Burge CB. 2004. Maximum entropy modeling of short sequence motifs with applications to RNA splicing signals. *J Comput Biol* **11**: 377-394.

Yeom KH, Pan Z, Lin CH, Lim HY, Xiao W, Xing Y, Black DL. 2021. Tracking pre-mRNA maturation across subcellular compartments identifies developmental gene regulation through intron retention and nuclear anchoring. *Genome Res* **31**: 1106-1119.

Yu KH, Shi CH, Wang B, Chow SH, Chung GT, Lung RW, Tan KE, Lim YY, Tsang AC, Lo KW et al. 2021. Quantifying full-length circular RNAs in cancer. *Genome Res* **31**: 2340-2353.

Zheng Y, Ji P, Chen S, Hou L, Zhao F. 2019. Reconstruction of full-length circular RNAs

enables isoform-level quantification. *Genome Med* **11**: 2.
